# Combined Patterns of IGHV Repertoire and Cytogenetic/Molecular Alterations in Monoclonal B Lymphocytosis versus Chronic Lymphocytic Leukemia

**DOI:** 10.1371/journal.pone.0067751

**Published:** 2013-07-03

**Authors:** Ana Henriques, Arancha Rodríguez-Caballero, Wendy G. Nieto, Anton W. Langerak, Ignacio Criado, Quentin Lécrevisse, Marcos González, Maria L. Pais, Artur Paiva, Julia Almeida, Alberto Orfao

**Affiliations:** 1 Cancer Research Center (IBMCC, USAL-CSIC), Department of Medicine and Cytometry Service, University of Salamanca (USAL) and Institute for Biomedical Research of Salamanca (IBSAL), Salamanca, Spain; 2 Blood and Transplantation Center of Coimbra/Portuguese Institute of Blood and Transplantation, IP, Coimbra, Portugal; 3 Department of Immunology, Erasmus MC, University Medical Center Rotterdam (Erasmus MC), Rotterdam, The Netherlands; 4 Service of Hematology, University Hospital of Salamanca, IBMCC, IBSAL and Department of Medicine, University of Salamanca, Salamanca, Spain; St. Jude Children’s Research Hospital, United States of America

## Abstract

**Background:**

Chronic lymphocytic leukemia (CLL)-like monoclonal B lymphocytosis (MBL) with (MBL^hi^) or without (MBL^lo^) absolute B-lymphocytosis precedes most CLL cases,the specific determinants for malignant progression remaining unknown.

**Methodology/Principal Findings:**

For this purpose, simultaneous iFISH and molecular analysis of well-established cytogenetic alterations of chromosomes 11, 12, 13, 14 and 17 together with the pattern of rearrangement of the IGHV genes were performed in CLL-like cells from MBL and CLL cases. Our results based on 78 CLL-like MBL and 117 CLL clones from 166 subjects living in the same geographical area, show the existence of three major groups of clones with distinct but partially overlapping patterns of IGHV gene usage, IGHV mutational status and cytogenetic alterations. These included a group enriched in MBL^lo^ clones expressing specific IGHV subgroups (e.g. VH3-23) with no or isolated good-prognosis cytogenetic alterations, a second group which mainly consisted of clinical MBL^hi^ and advanced stage CLL with a skewed but different CLL-associated IGHV gene repertoire (e.g. VH1-69), frequently associated with complex karyotypes and poor-prognosis cytogenetic alterations, and a third group of clones with intermediate features, with prevalence of mutated IGHV genes, and higher numbers of del(13q)^+^ clonal B-cells.

**Conclusions/Significance:**

These findings suggest that the specific IGHV repertoire and IGHV mutational status of CLL-like B-cell clones may modulate the type of cytogenetic alterations acquired, their rate of acquisition and/or potentially also their clinical consequences. Further long-term follow-up studies investigating the IGHV gene repertoire of MBL^lo^ clones in distinct geographic areas and microenvironments are required to confirm our findings and shed light on the potential role of some antigen-binding BCR specificities contributing to clonal evolution.

## Introduction

Monoclonal B lymphocytosis (MBL) is defined by the presence of a low to moderate expansion of circulating clonal B lymphocytes (<5×10^9^/L) –most frequently resembling the phenotype of chronic lymphocytic leukemia (CLL) cells (CLL-like cells)– in otherwise healthy adults, in the absence of symptoms and signs of an underlying chronic lymphoid malignancy [1], [2]. Recent multiparameter flow cytometry studies have demonstrated that CLL-like MBL clones can be found in a significant proportion of healthy adults over 40 years. Their frequency ranges from 3.5% to around 12% of the general population, and between 13% to 18% of first-degree relatives of CLL patients, depending on the sensitivity of the technique [3]. Although in most CLL-like MBL cases, MBL clones are associated with a stable and indolent clinical course, a small proportion of cases presenting with lymphocytosis will eventually progress to CLL [1], [4]. On the other hand, it has been shown that virtually every CLL is preceded by an MBL, which may have remained stable for variable periods of time [5]. Identification and full characterization of the phenotypic and genetic features of CLL-like MBL cells in the absence (MBL^lo^) and presence (MBL^hi^) of an absolute B-lymphocytosis, versus CLL cells, may provide insight into the key mechanisms and events involved in the expansion of the MBL clones and their transformation to CLL, thereby contributing to a better understanding of the natural history of the disease.

Previous studies have shown that MBL^hi^ clones may display the typical spectrum of chromosomal alterations observed in CLL, e.g. del(13q), trisomy 12, del(11q) and even del(17p); conversely, MBL^lo^ B-cells appear to more frequently carry normal karyotypes and to a lesser extent, isolated del(13q14.3) or trisomy 12, in the absence of chromosomal alterations associated to poor prognosis CLL, such as del(17p13) and del(11q22) [6]. These observations suggest that MBL^lo^, MBL^hi^ and CLL clones could be different stages in the spectrum from reactive MBL B-cells to CLL requiring therapy. Despite this, analysis of the pattern of usage of the immunoglobulin heavy chain variable region (IGHV) gene in both MBL^hi^ and CLL cases showed that it is not random. Accordingly, a predominant usage of specific IGHV subgroups has been reported for both MBL^hi^ clones and mutated CLL cells (e.g. the IGHV3-23 and IGHV4-34) as well as for unmutated CLL (e.g.IGHV1-69) [7]. In turn, very preliminary studies [8] indicate that MBL^lo^ clones rarely use the IGHV4-34 subgroup, while they may display a higher frequency of IGHV4-59/61 B-cell receptor (BCR) genes, which are rarely used in CLL [8].

Here, we investigated for the first time the potential existence of unique cytogenetic profiles associated with specific IGHV repertoires that could be associated with an increased risk of progression from MBL^lo^ to MBL^hi^ and CLL. Our results, based on a series of 78 MBL and 117 CLL clones from a total of 166 subjects from the same geographical area, suggest the existence of distinct but partially overlapping molecular and cytogenetic profiles among MBL^lo^, MBL^hi^ and CLL cases.

## Materials and Methods

### Patients and Samples

A total of 166 subjects presenting one or more CLL-like MBL and/or CLL clonal B-cell populations were included in this study: 15 cases (9%) corresponded to healthy individuals with MBL^lo^ – <200 clonal B-cells/µL in peripheral blood (PB); 5 males and 10 females; mean age of 68±13 years; range: 49–84 years, –33 (20%) were MBL^hi^ – ≥200 and <5,000 clonal CLL-like B-cells/µL of PB – (20 males and 13 females; mean age of 72±12 years; range: 37–89 years), –114 (69%) had newly-diagnosed untreated CLL (66 males and 48 females; mean age of 70±13 years; range: 35–89 years) and 4 (2%) had other B-cell lymphoproliferative disorders (B-CLPD) with coexistence of one or two CLL-like MBL B-cell population(s). From the 33 MBL^hi^ cases, 20 (61%) showed clinical MBL (>2,000 clonal B-cells/µl of PB). Individuals corresponded to consecutive MBL and CLL subjects from Salamanca (Spain) and Coimbra (Portugal) in the western area of the Iberian Peninsula.

PB samples were obtained from each subject after written informed consent was given, and the study was approved by the local ethics committees of the two participating centres (University Hospital of Salamanca and Histocompatibility Centre of Coimbra). Diagnosis of MBL and CLL was based on the World Health Organization (WHO) 2008 criteria [9]. Clinical staging of CLL subjects according to Binet classification [9] was collected retrospectively; 46/77 (60%) CLL cases were diagnosed as stage A and the remaining cases (31/77, 40%) as stage B/C. Overall, 37/166 subjects (22%) showed co-existence of two or three phenotypically different aberrant B-cell populations (multiclonal cases; 25 males and 12 females with a mean age of 76±8 years; range: 57–89 years), while the remaining 129 individuals showed one single phenotypically aberrant monoclonal B-cell population (monoclonal cases; 69 males and 60 females with a mean age of 68±12 years; range: 35–89 years). In 26/37 multiclonal cases, all different B-cell populations showed a typical CLL-like phenotype, while in the remaining 11 cases only one B-cell population displayed a typical CLL-like phenotype co-existing with population(s) phenotypically compatible with other B-CLPD [10]. For this study, analysis was focused only on those aberrant B-cell populations displaying a typical CLL-like and CLL phenotype (n = 195 B-cell clones). The distribution of all CLL-like and CLL clonal populations analyzed in the distinct diagnostic categories was as follows: 27 corresponded to CLL-like MBL^lo^, 51 to CLL-like MBL^hi^ and 117 to CLL (Table 1).

**Table 1:** Distribution of subjects included in the study and the corresponding CLL and CLL-like MBL clones, according to diagnosis.

			Diagnostic subgroups			
		No. of cases	MBL^lo^	MBL^hi^	CLL	Other B-CLPD
Subjects	Monoclonal	129	13 (87%)	25 (76%)	91 (80%)	–
	Multiclonal	37†	2 (13%)*	8 (23%)*	23 (20%)*	4 (100%)*
	**Total**	**166**	**15**	**33**	**114**	**4**
B-cell clones	From monoclonal cases	129	13 (48%)	25 (49%)	91 (78%)	–
	From multiclonal cases	66	14 (52%)	26 (51%)	26 (22%)	–
	***Total***	**195**	**27**	**51**	**117**	–

* For multiclonal CLL and CLL-like MBL cases as well as for other B-CLPD cases other than CLL, only CLL-like clones were considered; the later B-CLPD cases included the following diagnoses: HCL, hairy cell leukemia; SMZL/MALT, splenic marginal zone B-cell lymphoma/extranodal marginal zone B-cell lymphoma of mucosa-associated lymphoid tissue lymphoma. CLL, chronic lymphocytic leukemia; MBL, monoclonal B-cell lymphocytosis; B-CLPD, B-cell chronic lymphoproliferative disorders.

† The number of clones per multiclonal case was of two in all diagnostic subgroups, except in three tri-clonal subjects corresponding to one CLL patient, one MBL^hi^ case and one patient with a B-CLPD other than CLL.

### Immunophenotypic Analyses

Immunophenotypic studies were performed on erythrocyte-lysed whole PB samples according to procedures previously described in detail [11]. PB white blood cells (WBC) were systematically stained with the monoclonal antibody (MAb) combinations detailed in Table S1. All cases showed a clonal (imbalanced SmIgκ:SmIgλ ratio of >3∶1 or <1∶3) and/or an aberrant CD5^+^CLL-like B-cell population, as reported elsewhere [12] (see Materials and Methods S1).

Data acquisition was performed in two steps on a FACSCanto II flow cytometer – Becton-Dickinson Biosciences –(BD, San José, CA, USA) using the FACSDiva software (V6.1; BD): first, information about 1×10^5^ events corresponding to the whole sample cellularity was stored; in the second step, information was stored exclusively for CD19^+^ and/or CD20^+^gated events, contained in a minimum of 5×10^6^ leucocytes/tube. Instrument setup, calibration and daily monitoring were performed according to well-established protocols [13] (see Materials and Methods S1). For data analysis, the Infinicyt™ software (Cytognos SL, Salamanca, Spain), was used. The minimum number of clustered events required to define a B-cell population was of 50 cells.

### Fluorescence-activated Cell Sorting (FACS) Purification of B-cell Populations (FACSorting)

For all individuals studied, each CLL-like CD5^+^ B-cell population identified was purified in a FACSAria III flow cytometer (BD). In those samples with more than one aberrant B-cell population (n = 37), discrimination among them was based on their distinct patterns of expression for ≥1 of the B-cell markers analyzed, as described elsewhere [11]. The clonal nature of each FACS-purified B-cell population (purity: 98%±0.8%) was assessed by both cytogenetic and molecular techniques, as described below.

### Cytogenetic and Molecular Studies

The presence of the most common cytogenetic abnormalities associated with CLL was investigated by multicolour interphase fluorescence *in situ* hybridization (iFISH) on slides containing FACS-purified and fixed aberrant B-cells, as previously described [14] (see Materials and Methods S1 for further details).

Analysis of the patterns of rearrangement of the IGHV genes was performed for each FACS-purified CLL and CLL-like B-cell population. Genomic DNA preparation, PCR amplification, sequencing and analysis of V, (D), J genes were performed following well-established protocols [15], [16] (see Materials and Methods S1 for more detailed information).

Forward and reverse sequences were aligned into a single resolved sequence and then aligned with germline sequences using the IMGT database and tools (http://imgt.cines.fr). For MBL^lo^ clones, whole genomic amplification (WGA) was performed prior to the analysis, using the Replig^R^ UltraFast Mini kit (Qiagen, Valencia, CA) as per the recommendations of the manufacturer. For each clonal B-cell population, only in-frame rearrangements were evaluated. Sequences containing >2% deviation from the germline sequence were considered as being somatically mutated. Those MBL^lo^ cases showing more than one productive rearrangement corresponding to different IGHV genes within each purified CLL-like B-cell population were excluded from this study, because in such cases we could not establish the precise association between each IGHV gene and the underlying cytogenetic alterations detected.

Analysis of CLL-associated *NOTCH1* mutations [17] was performed via PCR of previously amplified genomic DNA from each FACS-purified CLL-like B-cell population for a total of 70 clones (5 MBL^lo^, 14 MBL^hi^ and 51 CLL clones).

### Statistical Methods

Conventional descriptive and comparative statistics –the nonparametric Kruskal-Wallis and Mann-Whitney U tests (for continuous variables), or the Pearson’s χ2 and Fisher exact tests (for categorical variables)–were performed using the SPSS software program (SPSS 15.0 Inc. Chicago, IL). *P* values <0.05 were considered to be associated with statistical significance.

For multivariate comparisons among MBL^lo^, MBL^hi^ and CLL clones, based on the count of clonal B cells/µL and the percentage of aberrant/clonal cells carrying the different cytogenetic profiles, principal component analysis (PCA) was applied, and graphically visualized with the 3D Automated Population Separator (APS) view – Principal Component 1 (PC1) vs PC2 vs PC3– of the Infinicyt™ software (Cytognos SL, Salamanca, Spain). As previously described in detail, in this APS view, each axis of a plot is represented by a different PC as a linear combination of parameters with distinct statistical weights [18].

For the assignment of MBL^lo^, MBL^hi^ and CLL clones to different groups, the size of the clone and the percentage of altered cells for each cytogenetic abnormality were the continuous variables included in the PCA-based assay performed with the Infinicyt software™, while IGHV gene usage, IGHV mutational status and clinical staging of CLL subjects according to the Binet classification were treated as categorical variables, used only for labelling the different clones within each group, after applying the PCA.

## Results

### Overall Size and BCR Features of CLL-like MBL and CLL B-cell Clones

The median relative percentage and absolute count of CLL-like and CLL B-cells progressively increased from MBL^lo^ (0.6% and 20 cells/µl), to MBL^hi^ (14% and 2,000 cells/µl) and CLL clones (57% and 17,400 cells/µl) (*P*<0.0001) (Table 2).

**Table 2:** Peripheral blood (PB) B-cell counts and BCR features of clonal MBL^lo^, MBL^hi^ andCLL B cells.

	MBL^lo^N = 27	MBL^hi^N = 51	CLLN = 117	*P* value
No. of PB clonal B cells(×10^6^/L)*	20 (0. 09–200)	2,000 (350–4,900)	17,400 (1,300†–369,000)	*P*<0.0001^a,b,c,d,e^
% of PB clonal B cells from WBC*	0.6% (0.001%–7.5%)	14% (0.7%–45%)	57% (17%–97%)	*P*<0.0001^a,b,c,d,e^
No. of B-cell clones from multiclonal cases	14/27 (52%)	26/51 (51%)	26/117(22%)	*P*≤0.002^a,b,d^;*P* = 0.03^e^
No. of IGHV mutated clones	18/27 (67%)	37/51 (73%)	60/113 (53%)	*P*≤0.02^a,d^

Results expressed as number of B-cell clones and percentage between brackets or as *median value (range). Statistically significant differences (*P*<0.05) found between ^a^MBL^hi^ vs CLL, ^b^MBL^lo^ vs CLL, ^c^MBL^lo^ vs MBL^hi^, ^d^MBL^lo^ plus MBL^hi^ vs CLL and ^e^MBL^lo^ vs MBL^hi^ plus CLL. BCR, B-cell receptor; CLL, chronic lymphocytic leukemia; MBL, monoclonal B-cell lymphocytosis; SmIg, surface membrane immunoglobulin; *IGHV*, immunoglobulin heavy chain variable region genes.

† Includes 6/117 cases with<5,000 clonal CLL B-cells/µL of PB, diagnosed with small lymphocytic lymphoma (SLL).

Of note, around half of all MBL^lo^ and MBL^hi^ cell populations (52% and 51%, respectively) derived from multiclonal cases, whereas only 22% of CLL clones were identified in multiclonal cases (*P*≤0.03; Table 2). In addition, CLL clones less frequently showed mutated *IGHV* genes (53%) compared to both MBL^hi^ (73%) and MBL^lo^ (67%) clones (*P*≤0.02) (Table 2).

### Cytogenetic Features and *NOTCH1* Mutation in CLL-like MBL and CLL B-cell Clones

Overall, MBL^lo^ B-cell clones showed a significantly lower frequency of genetic alterations associated with CLL (33%) than MBL^hi^ (51%) and CLL (62%) B-cells (*P*≤0.02) (Table 3). Furthermore, only a small proportion of MBL^lo^ (7%) and MBL^hi^ clones (14%) showed coexistence of ≥2 cytogenetic alterations, while this was found in 33% of all CLL clones (*P*≤0.04).

**Table 3:** Cytogenetic and molecular features of MBL^lo^, MBL^hi^ and CLL B-cell clones.

Cytogenetic/molecular alterations	MBL^lo^N = 27	MBL^hi^N = 51	CLLN = 117	*P* value
No. of genetically altered CLL-like/CLL clones	9/27 (33%)	26/51 (51%)	72/117 (62%)	*P*≤0.02^b,e^
No. of clones with ≥2 genetic alterations	2/27 (7%)	7/51 (14%)	38/117 (33%)^†^	*P*≤0.04^a,b,d^
Type of cytogenetic/molecular changes				
No. of del(13q)^+^ clones (%)% del(13q)^+^cells *	7/27 (26%)73% (19%–96%)	16/51 (31%)56% (15%–99%)	46/117 (39%)87% (10%–99%)	NSNS
No. of del(13q14.3)^+^ clones (%)% del(13q14.3)^+^cells *	5/27 (19%)70% (19%–96%)	16/51 (31%)46% (15%–99%)	45/117 (39%)75% (5%–99%)	NSNS
No. of del(13q14)^+^ clones (%)% del(13q14)^+^cells *	1/26 (4%)86% (86%–86%)	3/51 (6%)96% (15%–98%)	18/117 (15%)79% (10%–99%)	*P* = 0.04^d^NS
No. of trisomy 12^+^ clones (%)% trisomy 12^+^ cells *	2/27 (7%)50% (41%–59%)	10/51 (20%)87% (66%–95%)	20/117 (17%)77% (33%–97%)	NS*P*≤0.04^b,e^
No. of t(14q32)^+^ clones (%)% t(14q32)^+^ cells *	0/17 (0%)–	2/51 (4%)42% (31%–52%)	12/116 (10%)82% (18%–98%)	NSNS
				
No. of del(11q)^+^ clones (%)% del(11q)^+^cells *	0/23 (0%)–	2/51 (4%)57% (20%–93%)	9/116 (8%)58% (21%–98%)	NSNS
No. of del(11q22.3)^+^ clones (%)% del(11q22.3)^+^cells *	0/23 (0%)–	2/51 (4%)57% (20%–93%)	7/116 (6%)71% (24%–98%)	NSNS
No. of del(11q23)^+^ clones (%)% del(11q23)^+^cells *	0/15 (0%)–	0/51 (0%)–	4/116 (3%)32% (21%–64%)	NS–
No. of del(17p13.1)^+^ clones (%)% del(17p13.1)^+^cells *	0/24 (0%)–	0/51 (0%)–	5/117 (4%)44% (33%–88%)	NS–
No. of *NOTCH1* mutated clones (%)	0/5 (0%)	0/14 (0%)	5/52 (10%)	NS

Results expressed as number of B-cell clones with cytogenetic abnormalities from all clones in the corresponding group (percentage) or as *median values of altered cells/clone (range). In 9 clones (1 MBL^lo^,1MBL^hi^ and 7 CLL) biallelic del(13q14.3) was detected and hyperdiploidy was found in one MBL^lo^ clone. ^a^MBL^hi^ vs CLL, ^b^MBL^lo^ vs CLL, ^d^MBL^lo^ plus MBL^hi^ vs CLL and ^e^MBL^lo^ vs MBL^hi^ plus CLL. NS, no statistically significant differences observed (*P≥*0.05); CLL, chronic lymphocytic leukemia; MBL, monoclonal B-cell lymphocytosis.^†^Includes the 5/66 cases with *NOTCH1* mutation associated to trisomy 12 in 3 cases, to biallelicdel(13q14.3) in one and to both monoallelic del(13q14.3) and del(17p) in the remaining cases.

Regarding each specific cytogenetic/molecular alteration, a tendency towards a greater frequency of del(13q) and trisomy 12 was observed from MBL^lo^ to MBL^hi^ and CLL clones, although differences only reached statistical significance for the frequency of del(13q14) involving the *RB1* gene (*P* = 0.04). In addition, presence of t(14q32) and del(11q22.3) were exclusively found among MBL^hi^ and CLL, while del(17p), del(11q23) and *NOTCH1* mutations were only present in CLL clones (Table 3). Of note, among the cytogenetically altered clones, no significant differences were observed in the percentage of altered cells, except for a greater proportion of B-cells with trisomy 12 among both MBL^hi^ and CLL vs MBL^lo^ B-cell clones (*P*≤0.04) (Table 3).

*NOTCH1* mutations occurred in 5/52 CLL cases (10%), in which a preferential association with IGHV unmutated clones (80%, *P = *0.02) and a high frequency of cases (3/5, 60%) harbouring trisomy 12 as an additional isolated chromosomal abnormality (*P = *0.007) was observed; in the remaining two CLL cases, the presence of *NOTCH1* mutation was associated with del(13q14) involving the *RB1* gene and to both del(13q14.3) and del(17p), respectively. None of the 19 MBL cases investigated showed *NOTCH1* mutations.

### Molecular Characteristics of CLL-like MBL and CLL B-cell Clones

Analysis of the IGHV gene revealed single in frame gene rearrangements for each clonal B-cell population analyzed. Interestingly, shorter CDR3 sequences of the IGHV gene were found among MBL^lo^ versus CLL clones. So, the frequency of CLL clones with CDR3 sequences coding for >20 aminoacids became significantly higher than that observed among MBL^lo^ and MBL^hi^ clones (*P* = 0.02) (Table 4).

**Table 4:** Molecular characteristicsof the BCR of CLL-like MBL^lo^, MBL^hi^ and CLL B-cell clones.

	MBL^lo^N = 27	MBL^hi^N = 51	CLLN = 113	*P* value
HCDR3 length*	13 (6–22)	17 (8–26)	18 (8–32)	*P*≤0.02^b,d,e^
HCDR3>20 aa	3/27 (11%)	8/51 (16%)	38/113 (34%)	*P* = 0.02^a,b,d^
VH subgroupsVH1	4/27 (15%)	7/51 (15%)	28/113 (25%)	NS
VH2	0/27 (0%)	1/51 (2%)	2/113 (2%)	NS
VH3	18/27 (66%)	31/51 (60%)	47/113 (42%)	*P*≤0.04^a,b,d^
VH4	4/27 (15%)	9/51 (17%)	32/113 (28%)	*NS*
VH5	1/27 (4%)	3/51 (6%)	2/113 (2%)	NS
VH6	0/27 (0%)	0/51 (0%)	1/113 (1%)	NS
DH subgroupsDH1,4,7	5/27 (18.5%)	7/50 (14%)	15/109 (14%)	NS
DH2	5/27 (18.5%)	12/50 (24%)	19/109 (17%)	NS
DH3	5/27 (18.5%)	16/50 (32%)	48/109 (44%)	*P*≤0.03^b,d,e^
DH5	5/27 (18.5%)	8/50 (16%)	12/109 (11%)	NS
DH6	7/27 (26%)	7/50 (14%)	15/109 (14%)	NS
JH genesJH1,2,3,5	4/27 (15%)	14/50 (28%)	23/113 (20%)	NS
JH4	13/27 (48%)	21/50 (42%)	45/113 (40%)	NS
JH6	10/27 (37%)	15/50 (30%)	45/113 (40%)	NS

Results expressed as number of B-cell clones from all clones in the corresponding group (percentage) or or as *median (range). Statistically significant differences were found between ^a^MBL^hi^ vs CLL, ^b^MBL^lo^ vs CLL, ^c^MBL^lo^ vs MBL^hi^, ^d^ MBL^lo^ plus MBL^hi^ vs CLL and ^e^MBL^lo^ vs MBL^hi^ plus CLL; NS, no statistically significant differences observed (*P≥*0.05);BCR, B-cell receptor; CLL, chronic lymphocytic leukemia; MBL, monoclonal B-cell lymphocytosis; aa, aminoacids; HCDR3,heavy chain complementarity-determining region 3.

**Figure 1:**
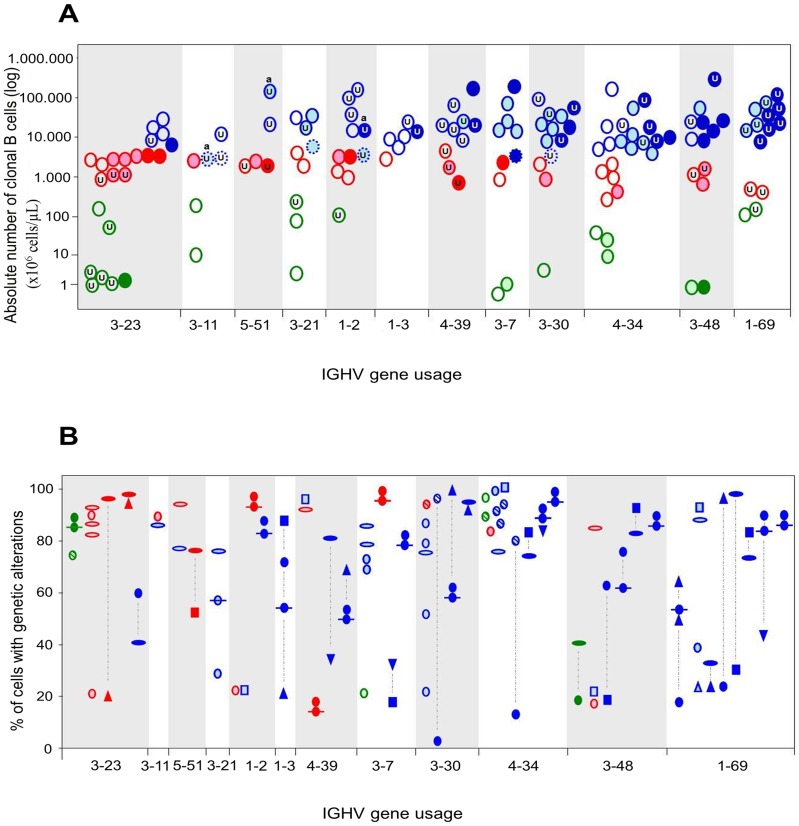
Frequency of CLL-associated cytogenetic alterations (A) and the cytogenetic profile (B) for those IGHV genes most commonly detected in “low-count MBL”(MBL^lo^), “high-count MBL” (MBL^hi^) and CLL B-cell clones, as assessed by interphase fluorescence in situ hybridization (iFISH). The three diagnostic categories studied are depicted by different colors (green, MBL^lo^; red, MBL^hi^; blue, CLL B-cell clones) and the absence vs presence of one vs≥2 chromosomal alterations*per*clone, is indicated by empty circles, light colored and dark colored circles, respectively. For each IGHV subgroup, the clones are represented in the Y-axis according to the absolute number of clonal B cells per µL of PB (**A**) and the percentage of cells genetically altered, by iFISH (**B**). Different FISH patterns are defined by the following symbols in panel **B**:, del(13q14.3);, biallelic del(13q14.3);, del(13q14);, trisomy 12; Δ, del(11q);**▿,**del(17p) and; **□**, t(14q32); dotted contour lines in panel **A** highlight those clones phenotypically classified as SLL(small lymphocytic lymphoma); dotted blue lines in panel **B** indicate cells from the same B-cell clone showing different cytogenetic abnormalities; U = unmutated clones; a = clones with *NOTCH1* mutation.

Regarding IGHV, DH and JH gene usage, no significant differences were found between the three groups of CLL-like B-cell clones, except for the VH3 and DH3 IGHV genes (Table 4): CLL showed lower frequency of VH3 usage and a greater proportion of DH3*^+^*clones vs MBL^lo^ and MBL^hi^ (*P*≤0.04) (Table 4). Of note, a significant percentage of all CLL (72%), MBL^hi^ (74%) and MBL^lo^ (85%) clones corresponded to only 12 IGHV genes, namely V3-23, V3-11, V5-51, V3-21, V1-2, V1-3, V4-39, V3-7, V3-30, V4-34, V3-48 and V1-69 (Figure 1). Among these, preferential usage (*P*<0.04) of the VH3-23 gene was observed in both MBL^lo^ (7/27, 26%) and MBL^hi^ (10/51, 20%) vs CLL (5/113, 4%) clones.

### Relationship between the Most Frequently used IGHV Genes and the Cytogenetic Profile of CLL-like MBL and CLL B-cell Clones

As mentioned above, preferential usage of the VH3-23 gene was observed in both MBL^lo^ and MBL^hi^ versus CLL clones (Figure 1A). VH3-23^+^ MBL clones typically showed no cytogenetic alterations (8/17) or they carried an isolated cytogenetic alteration which corresponded either to trisomy 12 (3/17) or deletion of 13q (3/17) (Figure 1B). Nevertheless, two MBL^hi^ clones showed co-existence of trisomy 12 and del(11q22.3) and one MBL^lo^ clone showed del(13q) including both the 13q14.3 and 13q14 (*RB1*) chromosomal regions (Figure 1B). From the fiveVH3-23^+^ CLL clones only one carried genetic alterations –trisomy 12 and del(13q) –. Most interestingly, VH3-23^+^MBL^lo^ clones frequently showed unmutated IGHV genes, including most unmutated MBL^lo^ clones, with <10 CLL-like cells/µl(5/8; 63%), which contrasts to the much lower frequency of unmutated VH3-23 CLL clones.

A similar frequency of usage of the VH3-11, VH5-51, VH3-21 and VH1-2 genes was observed in both MBL^lo^ and MBL^hi^ versus CLL (Figure 1). In none of the clones expressing these IGHV genes, cytogenetic alterations associated with a poor disease outcome – e.g. del(17p) and/or del(11q) – were found; in addition, most MBL and CLL clones expressing these IGHV genes showed no cytogenetic alteration, or they just had a single abnormality. Despite this, *NOTCH1* mutations were more frequently observed among cytogenetically altered, IGHV unmutated CLL clones expressing these IGHV genes (one VH3-11^+^, one VH3-21^+^ and one VH1-2^+^ clones). Noteworthy, 4/6 CLL cases classified as small lymphocytic lymphoma (SLL) variants were also included among cases with a VH3-11 (n = 2), VH3-21 (n = 1) and VH1-2 (n = 1) repertoire in this group.

Finally, among those clonal B-cell populations which expressed the VH1-3, VH4-39, VH3-7, VH3-30, VH4-34, VH3-48 and VH1-69 genes, CLL clones were overrepresented (61/113, 54%) versus both MBL^lo^ (10/27, 37%) and MBL^hi^ (18/51, 35%) clones. Notably, a high number of CLL clones carrying these IGHV genes in association with one or more cytogenetic alterations, including poor prognosis cytogenetic alterations, was found among these cases (44/61, 72%). In this regard, del(13q) including both the 13q14.3 and 13q14 (*RB1*) chromosomal regions was frequently detected (single or combined lesion) in these CLL and also MBL^hi^ clones, particularly among those cases expressing the VH3-30 and VH4-34 gene genes; presence of trisomy 12, del(11q) and t(14q32) were also common among these CLL cases (16%, 13% and 18%, respectively) while being infrequent in MBL cases (only 2 MBL^hi^ clones showed isolated trisomy 12). Moreover, del(17p) alone and complex karyotypes with ≥3 cytogenetic/molecular alterations were also found in 4 of the CLL clones which expressed the VH1-3, VH4-39, VH3-30, VH3-48, VH4-34 and VH1-69 IGH genes, respectively (Figure 1B). Remarkably, ≥1 genetic alteration was systematically detected in a major fraction of the VH1-69^+^ clonal cells (Figure 1A) while being absent in the few MBL clones which expressed this specific IGHV gene. Of note, unmutated IGHV genes were a hallmark of both VH1-69^+^ (12/14 clones; 86%) and VH4-39^+^ (9/10 clones; 90%), independently of their MBL vs CLL nature (Figure 1A).

**Figure 2:**
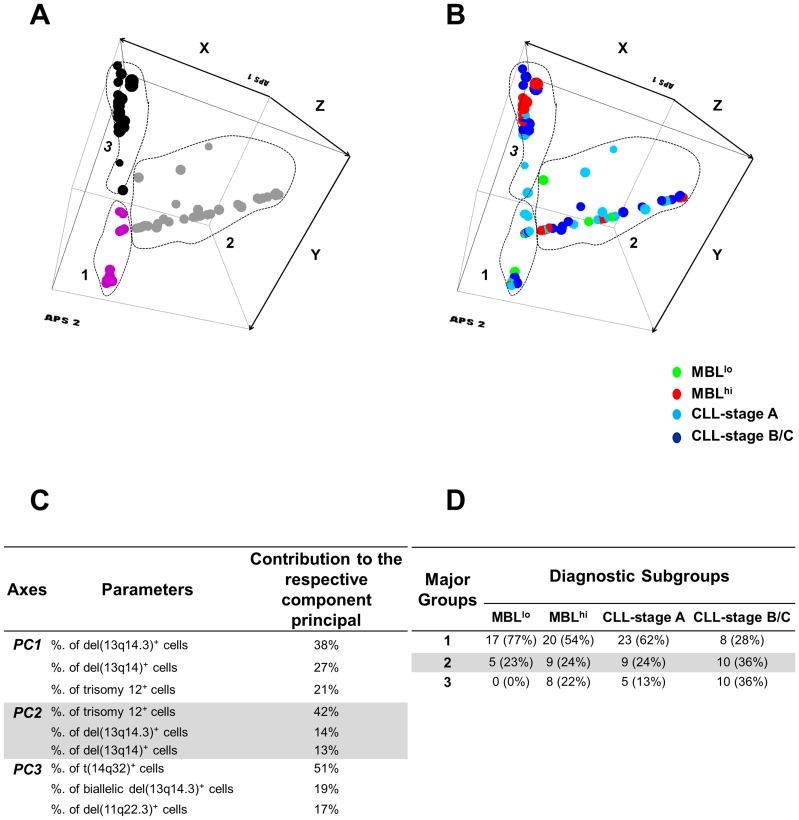
Principal component analysis (3-dimensionalX-Y-Z axis view of PC1 vs PC2 vs PC3, respectively) for comparison of “low-count MBL” (MBL^lo^), “high-count MBL” (MBL^hi^) and CLL B-cell clones according to the absolute number of clonal B cells/µL and the pattern of cytogenetic alterations (including the percentage of altered cells), using the Infinicyt^TM^software. Overall, MBL^lo^, MBL^hi^ and CLL cases are clustered into groups distinguished by different colors in A: magenta, gray, and black circles (**A**). The distribution of MBL^lo^, MBL^hi^, CLL-stage A and CLL-stage B/C clones are coloured differently in B: MBL^lo^, green; MBL^hi^, red, CLL stage A and B/C light blue and dark blue, respectively (**B**). The most informative parameters contributing to the best discrimination between 1×1 comparisons of the three groups are displayed in a decreasing order of percentage contribution to each of the principal component (**C**); Distribution of MBL^lo^, MBL^hi^ and CLL clones among the three major groups defined in panel A by principal component analysis (**D**); CLL, chronic lymphocytic leukemia; MBL, monoclonal B lymphocytosis; PC: principal component.

Based on the observation of the above described associations, we performed a multivariate analysis based on PCA, in searching for unique patterns of association between cytogenetic alterations and IGHV repertoires among MBL vs CLL clones. Three major groups of CLL-like MBL and CLL clones were identified, according to the absolute number of clonal B cells/µL and the percentage of cells carrying cytogenetic alterations (Figure 2) and then labelled according to their pattern of IGHV gene usage and the VH mutational status. Of note, the most homogeneous group (Group 1) included virtually all MBL^lo^ clones (77%) and half of the MBL^hi^ clones (54%), but only around one fourth of Binet stage B/C CLL (28%); by contrast, no MBL^lo^ clones were included in Group 3 (Figure 2D). Group 2 showed a more heterogeneous distribution with an intermediate pattern. In detail, Group 1 was mainly characterized by cases with a normal karyotype (83%) and lower numbers of cytogenetically altered cells mostly displaying the VH1-2, VH3-23 and VH4-34 IGHV genes (Table S2); in turn, Group 2 typically showed a higher number of cases with mutated IGHV genes, and higher numbers of del(13q)^+^ clonal B cells, while Group 3 included high numbers of cases with unmutated IGHV genes, trisomy 12 and an IGHV repertoire enriched in unmutated VH1-69^+^ CLL clones (Table S2).

## Discussion

It is now well established that emergence of CLL is typically preceded by MBL [5]. However, only a fraction of all MBL^hi^ will evolve to CLL, at a rate of 1.1% persons/year [19], while the outcome of MBL^lo^ remains unknown. Despite this, general consensus exists in that stepwise acquisition of specific genetic alterations may determine the rate of progression, not only from MBL^hi^ to CLL, but potentially also from MBL^lo^ to MBL^hi^ and eventually to CLL. Concurrence of chronic antigen stimulation through specific BCRs may further support and accelerate the expansion of MBL clones, facilitate acquisition of new genetic alterations and therefore contribute to progression to CLL [6], [20]. Although data has accumulated in the last decade about the cytogenetic alterations and the IGHV gene repertoire of CLL-like clonal B-cells in both MBL and CLL, to our knowledge, this is the first report about the combined patterns of cytogenetic alterations and IGHV gene repertoire in MBL^lo^ vs MBL^hi^ vs CLL clones.

In recent years, more than a thousand different molecular/genetic alterations reflected in multiple distinct and complex cytogenetic/molecular profiles in individual CLL patients, have been described through high-throughput whole-genome sequencing approaches [21], [22]. However, only a relatively small number of cytogenetic/molecular alterations recurrently occur at relatively high frequencies (e.g. >5% cases) [23], [24]. Such alterations include del(13q14), reported in around half of all CLL cases, trisomy 12, present in about one third of the patients and del(11q), del(17p), t(14q32) and *NOTCH1* mutations, which occur in between 5-15% of all CLL cases [14], [23], [24]. In around half of CLL cases, unmutated IGH genes associated with preferential usage of specific IGHV genes (i.e. VH1-69 and VH4-34) and the above described cytogenetic alterations have also been reported in CLL. In turn, MBL^hi^ cases share molecular features with good-prognosis CLL in terms of both the IGHV gene repertoire and chromosomal alterations [25], [26], with a greater frequency of IGHV mutated cases. By contrast, preliminary data indicates that the IGHV repertoire expressed by MBL^lo^ could be strikingly different from that of both typical CLL and MBL^hi^ cases [27]; in addition, such MBL^lo^ clones appear to display a much lower frequency of chromosomal alterations, restricted to del(13q14.3) and trisomy 12, with a high prevalence of IGHV mutated cases (similar to that of MBL^hi^ cases) [6], and no poor-prognosis cytogenetic alterations [8], [25].

In line with such observations, we also found a lower frequency of both cytogenetically altered and IGHV unmutated CLL-like clones in MBL^lo^ vs both MBL^hi^ and CLL and vs CLL clones, respectively. Interestingly, the proportion of B-cell clones carrying ≥2 alterations significantly increased from MBL^hi^ to CLL. On top of the progressively higher number of cytogenetic/molecular alterations found in MBL^lo^ vs MBL^hi^ and CLL, the cytogenetic profile of clonal B-cells also became significantly more heterogeneous among the latter two groups. Accordingly, while del(13q14.3) and to a much lesser extent, del(13q14) involving the *RB1* gene and trisomy 12, were already detected in a small fraction of MBL^lo^ clones, del(11q) and t(14q32) emerged at an MBL^hi^ stage, whereas del(17p), del(11q23) and *NOTCH1* mutations were only found in CLL. These latter three alterations typically involved CLL clones that already had other cytogenetic alterations and therefore, had more complex cytogenetic/molecular profiles. In line with these findings, the altered CLL-like MBL and CLL clones showed progressively increasing percentages of cells carrying del(13q14.3), del(13q14), trisomy 12, t(14q32),del(11q)and del(17p13.1),respectively. In accordance with previous observations [28], *NOTCH1* mutations were exclusively detected in CLL (preferentially among unmutated CLL clones) which also had other cytogenetic alterations – e.g., trisomy 12, del(13q14) and/or del(17p).

The overall increased frequency of all cytogenetic alterations, together with the more complex cytogenetic/molecular profiles, observed from MBL^lo^ to MBL^hi^ and CLL would support the notion that evolution from MBL^lo^ to MBL^hi^ and CLL is paralleled by progressive acquisition of recurrent cytogenetic alterations, each of which appears to emerge at specific MBL and CLL stages, in line with previous data from our and other groups [12], [29]. Accordingly, del(13q), and to a lesser extent trisomy 12, are relatively early cytogenetic events which may frequently occur at an MBL^lo^ stage, whereas del(17p), *NOTCH1* mutations, and to a lesser extent also del(11q) and t(14q32), would typically arise later, as secondary cytogenetic events occurring at an MBL^hi^ or CLL stage. Acquisition of these and other genetic changes may potentially be associated with an increased proliferation and/or survival of the altered CLL-like cells. At the earliest stages of development of MBL, proliferation and/or survival signals could be provided to the MBL clone by chronically sustained BCR stimulation. If this holds true, the BCR features could also play a critical role in determining the probability of cytogenetic progression. Unfortunately, our series of MBL -particularly of MBL^lo^- is quite short at this time to further confirm this hypothesis, due to the difficulty in collecting cases with enough number of CLL-like B-cells, to perform in parallel reliable iFISH and molecular analyses. In this regard, the limited number of MBL^lo^ cases included in our series may predominantly present with the genetic/molecular patterns of a low risk MBL cohort, which may not be related to CLL progression. Despite this, in accordance with other recent reports [1], [30], [31], non-random usage of IGHV genes with clearly different IGHV gene repertoires was found in our series in MBL vs CLL. As expected, the most frequently used IGHV genes in CLL were the VH4-34, VH3-30, VH1-69, VH3-48, VH4-39, VH1-2 and VH3-7 genes, accounting for around half of the CLL clones. Interestingly, also half of the CLL clones showed unmutated IGHV genes, strikingly high frequencies of unmutated clones being detected among cells expressing VH1-69, VH4-39 and VH1-2. By contrast, VH3-23^+^ B-cells predominated among the MBL^lo^ and MBL^hi^ clones, most VH3-23^+^ MBL^lo^ cases showing very low counts of IGHV unmutated clonal B-cells. Of note, the IGHV genes used by the MBL^hi^ clones were commonly observed in either CLL (e.g. VH4–34, VH1-2, VH3–48, and VH4–39) or MBL^lo^ (e.g. VH3-23 and VH4-34), but usually at lower frequencies. The fact that these particular IGHV genes have been associated with previously reported stereotypic B-cell receptors in CLL clones [32], together with our own results which show that the complementary-determining regions (CDR3) of the IGHV genes are highly homologous in around one fifth of the B-cell clones from our short cohort (Table S3), would reinforce the role of some antigen-binding BCR specificities in clonal evolution.

Based on the overall patterns of cytogenetic alterations and IGHV gene usage together with the BCR mutational status, it could be concluded that while some unmutated IGHV genes appear to be associated with the acquisition of complex cytogenetic profiles, rapid expansion of clonal CLL-like B-cells and progression to CLL (e.g. IGHV1-69), others would not (e.g. IGHV3-23); the latter clones would show a more benign behaviour. This could potentially be due to a lower binding affinity of the unmutated BCR for the antigen, the recognition of specific subtypes of low concentrated antigens and/or unique immune response profiles. In line with this hypothesis, IGHV genes over-represented among CLL clones (e.g.VH4-39 and VH1-69) frequently corresponded to IGHV genes enriched in genes encoding for antibodies that recognise a broad variety of relatively common and abundant (auto)antigens, including low-affinity BCR, e.g. myoglobulin, thyroglobulin, actin, and ssDNA [33], [34] associated with T-independent, type II autoimmune responses [35]. In contrast, the unmutated IGHV3-23 BCR was over-represented among our MBL^lo^ cases, normal peripheral blood IGHV3-23^+^ IgM^+^ B-cells being associated with recognition of superantigens [36]–[38]. Thus, the association between MBL^lo^ and unmutated IGHV3-23 could be potentially due to a low affinity of this particular BCR for low concentrated/prevalent (super)antigens, which would limit the development of repetitive immune responses associated with the expansion of MBL clones and/or their cytogenetic progression. A recent study [8] also reported MBL^lo^ cases to display an IGHV gene repertoire different from that of CLL patients (e.g. absence of IGHV1-69^+^ MBL^lo^ clones, together with a low frequency of the IGHV4-34 gene and overrepresentation of the IGHV4-59/61 genes); however, no preferential usage of the IGHV3-23 gene was found among MBL^lo^ cells in this series. Further studies investigating the IGHV gene repertoire of MBL^lo^ clones in distinct geographic areas and microenvironments, may shed light on those factors accounting for such apparent discrepancies, as an association between MBL^lo^ and previous history of infections has been recently reported in this setting [39].

Taken together, these results would support the notion that antigen-driven BCR-stimulation could be a triggering factor in driving CLL-like B-cells to expand, in line with recent data showing a significant association between MBL in the general population and the individual history of infectious diseases and vaccination [39], whilst depending on the nature of the antigenic stimuli, distinct patterns of cytogenetic changes might then occur. Thus, the specific combination of cytogenetic alterations acquired by the CLL-like B-cells may determine, for distinct antigenic stimuli, and specific BCR repertoires, the outcome of the genetically-targeted cell. Long-term longitudinal studies, ideally of the same cases at different time-points and at different stages of the disease, would be crucial to definitively confirm these hypotheses, although based on our preliminary follow-up date [40] this may require decades due to the stable nature of most MBL^lo^ clones in the short-term.

In summary, MBL and CLL clones appear to display a distinct but partially overlapping pattern of IGHV gene usage, IGHV mutational status and cytogenetic alterations, which may translate into distinct groups of clones with different genetic/molecular features associated with a distinct clinical behavior. Sequential studies in larger series of cases followed for long periods of time are ongoing to investigate the risk of progression and outcome of MBL clones with specific IGHV and iFISH cytogenetic profiles.

## Supporting Information

Table S1 Monoclonal antibody combinations used for the immunophenotypic analysis of CLL-like and CLL B cells.
(DOCX)

Table S2 Informative parameters of the CLL-like/CLL B-cell clones included in the three major groups graphically visualized with APS view of the Infinicyt™ software.
(DOCX)

Table S3 Heavy chain variable region (IGHV) sequences of CLL-like and CLL B-cell clones analyzed by the IMGT-V-QUEST tool.
(DOCX)

Materials and Methods S1 (DOCX)
