# Supplementary material for: Combined Patterns of IGHV Repertoire and Cytogenetic/Molecular Alterations in Monoclonal B Lymphocytosis versus Chronic Lymphocytic Leukemia
Source: PLoS One. 2013 Jul 3;8(7):e67751. doi: 10.1371/journal.pone.0067751 (PMC3701012; doi:10.1371/journal.pone.0067751)
Supplement: Table S1 — Monoclonal antibody combinations used for the immunophenotypic analysis of CLL-like and CLL B cells. (DOCX) [file pone.0067751.s001.docx]

**Supplemental data**

**Table S1.** **Monoclonal antibody combinations used for the immunophenotypic analysis of CLL-like and CLL B cells.**

| **Tube** | **PacB** | **PacO** | **FITC** | **PE** | **PerCPCy5.5** | **PECy7** | **APC** | **AF700** |
| --- | --- | --- | --- | --- | --- | --- | --- | --- |
| 1 | CD20 | CD45 | CD8 +  Anti-SmIgλ | CD56 + Anti-SmIgκ | CD4 | CD19 | CD3 | CD38 |
| 2 | CD20 | CD45 | CyBcl2 | CD23 | CD19 | CD10 | CD5 | CD38 |
| 3 | CD20 | - | Anti-SmIgλ | Anti-SmIgκ | CD19 | CD10 | CD5 | - |
| 4 | CD20 | - | CD22 | CCR6 | - | CD19 | CD5 | - |
| 5 | CD20 | - | CD103 | CD25 | CD5 | CD19 | CD11c | - |
| 6 | CD20 | - | CD43 | CD79b | CD5 | CD19 | CD49d | - |
| 7 | CD20 | - | SmIgM | CD27 | - | CD19 | CD5 | - |
| 8 | CD20 | - | FMC7 | CD24 | - | CD19 | CD5 | - |
| 9 | CD20 | - | CD3 | CyZap70 | - | CD19 | CD5 | - |

All reagents were purchased from Becton Dickinson Biosciences (BD; San José, CA; USA), except CD19-PECy7 which was obtained from Beckman/Coulter (Miami, FL. USA), CD20-PacB that was purchased from eBiosciences (San Diego, CA, USA), CD38-AF700 that was from Exbio (Prague, Czech Republic), CD45-PacO from Invitrogen (Carlsbad, CA, USA), CD79b-PE, CD24-PE, and CD43-FITC, that were from Immunotech (Marseille, France) and anti-bcl2-FITC, anti-IgM-FITC, anti-Igλ-FITC and anti-Igκ-PE, which were obtained from DAKO (Glostrup, Denmark). For the staining of Cybcl2 and CyZap70, the Fix and Perm^TM^ reagent kit (Invitrogen) was used, following the recommendations of the manufacturer. AF, Alexa Fluor; APC, Allophycocyanin; Cy, cytoplasmic; Cy7, cyanin7; FITC, fluorescein isothiocyanate;PacB, Pacific Blue; PacO, Pacific Orange; PE, phycoerythrin; PerCPCy5.5, peridinin–chlorophyll–protein–cyanin 5.5; Sm, surface membrane.
