# Supplementary material for: Combined Patterns of IGHV Repertoire and Cytogenetic/Molecular Alterations in Monoclonal B Lymphocytosis versus Chronic Lymphocytic Leukemia
Source: PLoS One. 2013 Jul 3;8(7):e67751. doi: 10.1371/journal.pone.0067751 (PMC3701012; doi:10.1371/journal.pone.0067751)
Supplement: Table S2 — Informative parameters of the CLL-like/CLL B-cell clones included in the three major groups graphically visualized with APS view of the Infinicyt™ software. (DOCX) [file pone.0067751.s002.docx]

**Table S2.** **Informative parameters of the CLL-like / CLL B-cell clones included in the three major groups graphically visualized with APS view of the Infinicyt^TM^ software**.

|  |  | Continuous parameters included for multivariate analysis based on PCA | | | | | | | | |  |  |  | |
| --- | --- | --- | --- | --- | --- | --- | --- | --- | --- | --- | --- | --- | --- | --- |
| Groups | ID | Percentage of del(13q14.3)^+^ cells | Percentage of biallelic del(13q14.3)^+^ cells | Percentage of del(13q14)^+^ cells | Percentage of trisomy 12^+^ cells | Percentage of t(14q32)^+^ cells | Percentage of del(11q22.3)^+^ cells | Percentage of del(11q23.3)^+^ cells | Percentage of del(17p13.1)^+^ cells | Absolute number of clonal B cells/µL | IGHV gene repertoire | IGHV mutational status* | Binet Stage** |  |
| 1 | 127_MBLo_V3-23_UM_del13q.xlsx | 0 | 73 | 0 | 0 | 0 | 0 | 0 | 0 | 54 | 3-23 | >98% |  |  |
| 1 | 134_MBLlo_V3-23_UM_0 | 0 | 0 | 0 | 0 | 0 | 0 | 0 | 0 | 0.11 | 3-23 | >98% |  |  |
| 1 | 135_MBLlo_V3-23_UM_0 | 0 | 0 | 0 | 0 | 0 | 0 | 0 | 0 | 0.17 | 3-23 | >98% |  |  |
| 1 | 136_MBLlo_V3-23_UM_0 | 0 | 0 | 0 | 0 | 0 | 0 | 0 | 0 | 0.47 | 3-23 | >98% |  |  |
| 1 | 137_MBLlo_V3-23_UM_0 | 0 | 0 | 0 | 0 | 0 | 0 | 0 | 0 | 0.57 | 3-23 | >98% |  |  |
| 1 | 41_MBLo_V3-21_UM_0.xlsx | 0 | 0 | 0 | 0 | 0 | 0 | 0 | 0 | 80 | 3-21 | >98% |  |  |
| 1 | 42_MBLo_V3-21_M_0.xlsx | 0 | 0 | 0 | 0 | 0 | 0 | 0 | 0 | 250 | 3-21 | <98% |  |  |
| 1 | 138_MBLlo_V3-21_M_0 | 0 | 0 | 0 | 0 | 0 | 0 | 0 | 0 | 3.05 | 3-21 | <98% |  |  |
| 1 | 77_MBLo_V4-34_M_0.xlsx | 0 | 0 | 0 | 0 | 0 | 0 | 0 | 0 | 39 | 4-34 | <98% |  |  |
| 1 | 140_MBLlo_V4-34_M_bidel13q | 0 | 91 | 0 | 0 | 0 | 0 | 0 | 0 | 9.31 | 4-34 | <98% |  |  |
| 1 | 35_MBLo_V3-11_M_0.xlsx | 0 | 0 | 0 | 0 | 0 | 0 | 0 | 0 | 10 | 3-11 | <98% |  |  |
| 1 | 36_MBLo_V3-11_M_0.xlsx | 0 | 0 | 0 | 0 | 0 | 0 | 0 | 0 | 200 | 3-11 | <98% |  |  |
| 1 | 93_MBLo_V1-69_M_0.xlsx | 0 | 0 | 0 | 0 | 0 | 0 | 0 | 0 | 115 | 1-69 | <98% |  |  |
| 1 | 94_MBLo_V1-69_UM_0.xlsx | 0 | 0 | 0 | 0 | 0 | 0 | 0 | 0 | 158 | 1-69 | >98% |  |  |
| 1 | 48_MBLo_V1-2_UM_0.xlsx | 0 | 0 | 0 | 0 | 0 | 0 | 0 | 0 | 112 | 1-2 | >98% |  |  |
| 1 | 69_MBLo_V3-7_M_0.xlsx | 0 | 0 | 0 | 0 | 0 | 0 | 0 | 0 | 0.09 | 3-7 | <98% |  |  |
| 1 | 72_MBLo_V3-30_M_0.xlsx | 0 | 0 | 0 | 0 | 0 | 0 | 0 | 0 | 3 | 3-30 | <98% |  |  |
| 1 | 26_MBhi_V3-23_M_0.xlsx | 0 | 0 | 0 | 0 | 0 | 0 | 0 | 0 | 986 | 3-23 | <98% |  |  |
| 1 | 27bi_MBhi_V3-23_UM_0.xlsx | 0 | 0 | 0 | 0 | 0 | 0 | 0 | 0 | 2900 | 3-23 | >98% |  |  |
| 1 | 28_MBhi_V3-23_M_0.xlsx | 0 | 0 | 0 | 0 | 0 | 0 | 0 | 0 | 3281 | 3-23 | <98% |  |  |
| 1 | 43bi_MBhi_V3_21_M_0.xlsx | 0 | 0 | 0 | 0 | 0 | 0 | 0 | 0 | 2078 | 3-21 | <98% |  |  |
| 1 | 44_MBhi_V3-21_M_0.xlsx | 0 | 0 | 0 | 0 | 0 | 0 | 0 | 0 | 4424 | 3-21 | <98% |  |  |
| 1 | 78bi_MBhi_V4-34_M_0.xlsx | 0 | 0 | 0 | 0 | 0 | 0 | 0 | 0 | 346 | 4-34 | <98% |  |  |
| 1 | 79_MBhi_V4-34_M_0.xlsx | 0 | 0 | 0 | 0 | 0 | 0 | 0 | 0 | 1352 | 4-34 | <98% |  |  |
| 1 | 80bi_MBhi_V4-34_M_0.xlsx | 0 | 0 | 0 | 0 | 0 | 0 | 0 | 0 | 2047 | 4-34 | <98% |  |  |
| 1 | 81_MBhi_V4-34_M_0.xlsx | 0 | 0 | 0 | 0 | 0 | 0 | 0 | 0 | 2311 | 4-34 | <98% |  |  |
| 1 | 95bi_MBhi_V1-69_UM_0.xlsx | 0 | 0 | 0 | 0 | 0 | 0 | 0 | 0 | 430 | 1-69 | >98% |  |  |
| 1 | 96bi_MBhi_V1-69_UM_0.xlsx | 0 | 0 | 0 | 0 | 0 | 0 | 0 | 0 | 511 | 1-69 | >98% |  |  |
| 1 | 49_MBhi_V1-2_M_0.xlsx | 0 | 0 | 0 | 0 | 0 | 0 | 0 | 0 | 999 | 1-2 | <98% |  |  |
| 1 | 50_MBhi_V1-2_M_0.xlsx | 0 | 0 | 0 | 0 | 0 | 0 | 0 | 0 | 1454 | 1-2 | <98% |  |  |
| 1 | 70_MBhi_V3-7_M_0.xlsx | 0 | 0 | 0 | 0 | 0 | 0 | 0 | 0 | 1264 | 3-7 | <98% |  |  |
| 1 | 73_MBhi_V3-30_M_0.xlsx | 0 | 0 | 0 | 0 | 0 | 0 | 0 | 0 | 3986 | 3-30 | <98% |  |  |
| 1 | 128bi_MBhi_V3-30_M_del13q.xlsx | 0 | 95 | 0 | 0 | 0 | 0 | 0 | 0 | 914 | 3-30 | <98% |  |  |
| 1 | 57bi_MBhi_V1-3_UM_0.xlsx | 0 | 0 | 0 | 0 | 0 | 0 | 0 | 0 | 3458 | 1-3 | >98% |  |  |
| 1 | 89_MBhi_V3-48_UM_0.xlsx | 0 | 0 | 0 | 0 | 0 | 0 | 0 | 0 | 1170 | 3-48 | >98% |  |  |
| 1 | 63_MBhi_V4-39_UM_0.xlsx | 0 | 0 | 0 | 0 | 0 | 0 | 0 | 0 | 4594 | 4-39 | >98% |  |  |
| 1 | 39_MBhi_V5-51_UM_0.xlsx | 0 | 0 | 0 | 0 | 0 | 0 | 0 | 0 | 2000 | 5-51 | >98% |  |  |
| 1 | 31_CLL_V3-23_UM_0_A.xlsx | 0 | 0 | 0 | 0 | 0 | 0 | 0 | 0 | 5200 | 3-23 | >98% | A |  |
| 1 | 32bi_CLL_V3-23_M_0_A.xlsx | 0 | 0 | 0 | 0 | 0 | 0 | 0 | 0 | 5680 | 3-23 | <98% | A |  |
| 1 | 33_CLL_V3-23_M_0_A.xlsx | 0 | 0 | 0 | 0 | 0 | 0 | 0 | 0 | 17488 | 3-23 | <98% | A |  |
| 1 | 34_CLL_V3-23_M_0_A.xlsx | 0 | 0 | 0 | 0 | 0 | 0 | 0 | 0 | 28710 | 3-23 | <98% | A |  |
| 1 | 47_CLL_V3-21_M_0_A.xlsx | 0 | 0 | 0 | 0 | 0 | 0 | 0 | 0 | 37985 | 3-21 | <98% | A |  |
| 1 | 83_CLL_V4-34_M_0_A.xlsx | 0 | 0 | 0 | 0 | 0 | 0 | 0 | 0 | 7103 | 4-34 | <98% | A |  |
| 1 | 84bi_CLL_V4-34_M_0_A.xlsx | 0 | 0 | 0 | 0 | 0 | 0 | 0 | 0 | 7597 | 4-34 | <98% | A |  |
| 1 | 85_CLL_V4-34_M_0_A.xlsx | 0 | 0 | 0 | 0 | 0 | 0 | 0 | 0 | 8653 | 4-34 | <98% | A |  |
| 1 | 130bi_CLL_V4-34_M_del13q_A.xlsx | 0 | 96 | 0 | 0 | 0 | 0 | 0 | 0 | 8382 | 4-34 | <98% | A |  |
| 1 | 131bi_CLL_V4-34_M_del13q_A.xlsx | 0 | 91 | 0 | 0 | 0 | 0 | 0 | 0 | 8398 | 4-34 | <98% | A |  |
| 1 | 132_CLL_V4-34_M_2del13q_A.xlsx | 14 | 79 | 0 | 0 | 0 | 0 | 0 | 0 | 11523 | 4-34 | <98% | A |  |
| 1 | 37_SLL_V3-11_UM_0_A.xlsx | 0 | 0 | 0 | 0 | 0 | 0 | 0 | 0 | 2404 | 3-11 | >98% | A |  |
| 1 | 38_CLL_V3-11_UM_0_A.xlsx | 0 | 0 | 0 | 0 | 0 | 0 | 0 | 0 | 14076 | 3-11 | >98% | A |  |
| 1 | 98_CLL_V1-69_UM_del11q_A.xlsx | 0 | 0 | 0 | 0 | 0 | 0 | 24 | 0 | 20880 | 1-69 | >98% | A |  |
| 1 | 56_CLL_V1-2_M_0_A.xlsx | 0 | 0 | 0 | 0 | 0 | 0 | 0 | 0 | 9524 | 1-2 | <98% | A |  |
| 1 | 71_CLL_V3-7_M_t14q_del17p_A.xlsx | 0 | 0 | 0 | 0 | 18 | 0 | 0 | 33 | 3055 | 3-7 | <98% | A |  |
| 1 | 58_CLL_V1-3_M_0_A.xlsx | 0 | 0 | 0 | 0 | 0 | 0 | 0 | 0 | 7220 | 1-3 | <98% | A |  |
| 1 | 59bi_CLL_V1-3_M_0_A.xlsx | 0 | 0 | 0 | 0 | 0 | 0 | 0 | 0 | 7833 | 1-3 | <98% | A |  |
| 1 | 61_CLL_V1-3_UM_0_A.xlsx | 0 | 0 | 0 | 0 | 0 | 0 | 0 | 0 | 20229 | 1-3 | >98% | A |  |
| 1 | 92_CLL_V3-48_M_t14q_A.xlsx | 0 | 0 | 0 | 0 | 22 | 0 | 0 | 0 | 64726 | 3-48 | <98% | A |  |
| 1 | 64bi_CLL_V4-39_UM_0_A.xlsx | 0 | 0 | 0 | 0 | 0 | 0 | 0 | 0 | 6800 | 4-39 | >98% | A |  |
| 1 | 65_CLL_V4-39_UM_0_A.xlsx | 0 | 0 | 0 | 0 | 0 | 0 | 0 | 0 | 17214 | 4-39 | >98% | A |  |
| 1 | 68bi_CLL_V4-39_UM_0_A.xlsx | 0 | 0 | 0 | 0 | 0 | 0 | 0 | 0 | 71485 | 4-39 | >98% | A |  |
| 1 | 87_CLL_V4-34_M_0_B.xlsx | 0 | 0 | 0 | 0 | 0 | 0 | 0 | 0 | 186012 | 4-34 | <98% | B |  |
| 1 | 133bi_CLL_V4-34_UM_del13q_C.xlsx | 0 | 85 | 0 | 0 | 0 | 0 | 0 | 0 | 66142 | 4-34 | >98% | C |  |
| 1 | 53_CLL_V1-2_UM_0_B.xlsx | 0 | 0 | 0 | 0 | 0 | 0 | 0 | 0 | 45081 | 1-2 | >98% | B |  |
| 1 | 54_CLL_V1-2_UM_0_B.xlsx | 0 | 0 | 0 | 0 | 0 | 0 | 0 | 0 | 120102 | 1-2 | >98% | B |  |
| 1 | 74_SLL_V3-30_UM_0_B.xlsx | 0 | 0 | 0 | 0 | 0 | 0 | 0 | 0 | 3509 | 3-30 | >98% | B |  |
| 1 | 76bi_CLL_V3-30_UM_0_B.xlsx | 0 | 0 | 0 | 0 | 0 | 0 | 0 | 0 | 49600 | 3-30 | >98% | B |  |
| 1 | 129_CLL_V3-30_M_2del13q_C.xlsx | 5 | 95 | 0 | 0 | 0 | 0 | 0 | 0 | 17175 | 3-30 | <98% | C |  |
| 1 | 66_CLL_V4-39_UM_0_B.xlsx | 0 | 0 | 0 | 0 | 0 | 0 | 0 | 0 | 22704 | 4-39 | >98% | B |  |
| 2 | 24_MBLo_V3-23_M_2del13q.xlsx | 86 | 0 | 86 | 0 | 0 | 0 | 0 | 0 | 1 | 3-23 | <98% |  |  |
| 2 | 115_MBLo_V4-34_M_del13q.xlsx | 96 | 0 | 0 | 0 | 0 | 0 | 0 | 0 | 26 | 4-34 | <98% |  |  |
| 2 | 139_MBLlo_V3-7_M_del13q | 22 | 0 | 0 | 0 | 0 | 0 | 0 | 0 | 0.4 | 3-7 | <98% |  |  |
| 2 | 141_MBLlo_V3-48_M_del13q_tris12 | 19 | 0 | 0 | 41 | 0 | 0 | 0 | 0 | 0.57 | 3-48 | <98% |  |  |
| 2 | 142_MBLlo_V3-48_M_del13q | 70 | 0 | 0 | 0 | 0 | 0 | 0 | 0 | 0.57 | 3-48 | <98% |  |  |
| 2 | 29_MBhi_V3-23_M_del13q.xlsx | 21 | 0 | 0 | 0 | 0 | 0 | 0 | 0 | 4040 | 3-23 | <98% |  |  |
| 2 | 101_MBhi_V3-23_M_del13q.xlsx | 88 | 0 | 0 | 0 | 0 | 0 | 0 | 0 | 3044 | 3-23 | <98% |  |  |
| 2 | 116bi_MBhi_V4-34_M_del13q.xlsx | 83 | 0 | 0 | 0 | 0 | 0 | 0 | 0 | 551 | 4-34 | <98% |  |  |
| 2 | 102bi_MBhi_V3-11_M_del13q.xlsx | 87 | 0 | 0 | 0 | 0 | 0 | 0 | 0 | 2692 | 3-11 | <98% |  |  |
| 2 | 51_MBhi_V1-2_M_del13q.xlsx | 23 | 0 | 0 | 0 | 0 | 0 | 0 | 0 | 4623 | 1-2 | <98% |  |  |
| 2 | 103_MBhi_V1-2_M_2del13q.xlsx | 96 | 0 | 96 | 0 | 0 | 0 | 0 | 0 | 4371 | 1-2 | <98% |  |  |
| 2 | 107bi_MBhi_V3-7_M_2del13q.xlsx | 98 | 0 | 98 | 0 | 0 | 0 | 0 | 0 | 2750 | 3-7 | <98% |  |  |
| 2 | 88bi_MBhi_V3-48_M_del13q.xlsx | 18 | 0 | 0 | 0 | 0 | 0 | 0 | 0 | 680 | 3-48 | <98% |  |  |
| 2 | 62bi_MBhi_V4-39_UM_2del13q.xlsx | 15 | 0 | 15 | 0 | 0 | 0 | 0 | 0 | 3174 | 4-39 | >98% |  |  |
| 2 | 118_CLL_V4-34_M_del13q_A.xlsx | 99 | 0 | 0 | 0 | 0 | 0 | 0 | 0 | 13167 | 4-34 | <98% | A |  |
| 2 | 119_CLL_V4-34_M_2del13q_A.xlsx | 97 | 0 | 95 | 0 | 0 | 0 | 0 | 0 | 105594 | 4-34 | <98% | A |  |
| 2 | 104_CLL_V1-2_UM_2del13q_A.xlsx | 84 | 0 | 93 | 0 | 0 | 0 | 0 | 0 | 7683 | 1-2 | >98% | A |  |
| 2 | 111bi_CLL_V3-30_M_del13q_A.xlsx | 80 | 0 | 0 | 0 | 0 | 0 | 0 | 0 | 7920 | 3-30 | <98% | A |  |
| 2 | 112_CLL_V3-30_M_del13q_A.xlsx | 53 | 0 | 0 | 0 | 0 | 0 | 0 | 0 | 13524 | 3-30 | <98% | A |  |
| 2 | 113_CLL_V3-30_UM_2del13q_del11q_A.xlsx | 60 | 0 | 60 | 0 | 0 | 98 | 0 | 0 | 32063 | 3-30 | >98% | A |  |
| 2 | 114_CLL_V3-30_M_del13q_A.xlsx | 88 | 0 | 0 | 0 | 0 | 0 | 0 | 0 | 33146 | 3-30 | <98% | A |  |
| 2 | 105_CLL_V1-3_UM_2del13q_t14q_del11q_A.xlsx | 73 | 0 | 58 | 0 | 86 | 0 | 21 | 0 | 11419 | 1-3 | >98% | A |  |
| 2 | 123_CLL_V3-48_UM_2del13q_2del11q_A.xlsx | 19 | 0 | 55 | 0 | 0 | 51 | 64 | 0 | 369289 | 3-48 | >98% | A |  |
| 2 | 45_CLL_V3-21_M_del13q_B.xlsx | 0 | 0 | 61 | 0 | 0 | 0 | 0 | 0 | 5314 | 3-21 | <98% | B |  |
| 2 | 46bi_CLL_V3-21_M_del13q_C.xlsx | 30 | 0 | 0 | 0 | 0 | 0 | 0 | 0 | 15309 | 3-21 | <98% | C |  |
| 2 | 97_CLL_V1-69_UM_del13q_del11q_B.xlsx | 25 | 0 | 0 | 0 | 0 | 95 | 0 | 0 | 17445 | 1-69 | >98% | B |  |
| 2 | 125_CLL_V1-69_UM_2del13q_del17p_C.xlsx | 91 | 0 | 93 | 0 | 0 | 0 | 0 | 44 | 23134 | 1-69 | >98% | C |  |
| 2 | 126_CLL_V1-69_M_del13q_C.xlsx | 40 | 0 | 0 | 0 | 0 | 0 | 0 | 0 | 45737 | 1-69 | <98% | C |  |
| 2 | 110_CLL_V3-7_M_2del13q_B.xlsx | 81 | 0 | 81 | 0 | 0 | 0 | 0 | 0 | 237817 | 3-7 | <98% | B |  |
| 2 | 120_CLL_V3-48_M_del13q_t14q_C.xlsx | 64 | 0 | 0 | 0 | 19 | 0 | 0 | 0 | 16670 | 3-48 | <98% | C |  |
| 2 | 121_CLL_V3-48_M_2del13q_C.xlsx | 77 | 0 | 66 | 0 | 0 | 0 | 0 | 0 | 26880 | 3-48 | <98% | C |  |
| 2 | 122bi_CLL_V3-48_M_2del13q_C.xlsx | 87 | 0 | 95 | 0 | 0 | 0 | 0 | 0 | 27200 | 3-48 | <98% | C |  |
| 2 | 106_CLL_V4-39_M_2del13q_del11q_C.xlsx | 51 | 0 | 47 | 0 | 0 | 68 | 0 | 0 | 190555 | 4-39 | <98% | C |  |
| 3 | 2_MBhi_V3-23_M_tris12.xlsx | 0 | 0 | 0 | 91 | 0 | 0 | 0 | 0 | 1358 | 3-23 | <98% |  |  |
| 3 | 3bi_MBhi_V3-23_UM_tris12.xlsx | 0 | 0 | 0 | 87 | 0 | 0 | 0 | 0 | 3360 | 3-23 | >98% |  |  |
| 3 | 4bi_MBhi_V3-23_M_tris12_del11q.xlsx | 0 | 0 | 0 | 95 | 0 | 93 | 0 | 0 | 3443 | 3-23 | <98% |  |  |
| 3 | 5_MBhi_V3-23_M_tris12_del11q.xlsx | 0 | 0 | 0 | 93 | 0 | 20 | 0 | 0 | 4851 | 3-23 | <98% |  |  |
| 3 | 19_MBhi_V3-48_UM_tris12.xlsx | 0 | 0 | 0 | 84 | 0 | 0 | 0 | 0 | 1661 | 3-48 | >98% |  |  |
| 3 | 11bi_MBhi_V4-39_UM_del13q.xlsx | 0 | 0 | 0 | 93 | 0 | 0 | 0 | 0 | 700 | 4-39 | >98% |  |  |
| 3 | 7bi_MBhi_V5-51_UM_tris12_t14q.xlsx | 0 | 0 | 0 | 76 | 52 | 0 | 0 | 0 | 1900 | 5-51 | >98% |  |  |
| 3 | 8bi_MBhi_V5-51_M_tris12.xlsx | 0 | 0 | 0 | 93 | 0 | 0 | 0 | 0 | 2659 | 5-51 | <98% |  |  |
| 3 | 10_CLL_V3-21_UM_tris12_A.xlsx | 0 | 0 | 0 | 75 | 0 | 0 | 0 | 0 | 30739 | 3-21 | >98% | A |  |
| 3 | 17bi_CLL_V4-34_M_tris12_A.xlsx | 0 | 0 | 0 | 75 | 0 | 0 | 0 | 0 | 5482 | 4-34 | <98% | A |  |
| 3 | 21_CLL_V1-69_UM_tris12_A.xlsx | 0 | 0 | 0 | 87 | 0 | 0 | 0 | 0 | 14470 | 1-69 | >98% | A |  |
| 3 | 99_CLL_V1-69_UM_tris12_del11q_A.xlsx | 0 | 0 | 0 | 33 | 0 | 24 | 0 | 0 | 30093 | 1-69 | >98% | A |  |
| 3 | 100_CLL_V1-69_UM_t14q_A.xlsx | 0 | 0 | 0 | 0 | 91 | 0 | 0 | 0 | 75170 | 1-69 | >98% | A |  |
| 3 | 22_CLL_V1-69_UM_tris12_t14q_B.xlsx | 0 | 0 | 0 | 73 | 82 | 0 | 0 | 0 | 46393 | 1-69 | >98% | B |  |
| 3 | 23_CLL_V1-69_UM_tris12_t14q_B.xlsx | 0 | 0 | 0 | 97 | 30 | 0 | 0 | 0 | 119647 | 1-69 | >98% | B |  |
| 3 | 6_SLL_V3-11_UM_tris12_B.xlsx | 0 | 0 | 0 | 84 | 0 | 0 | 0 | 0 | 2531 | 3-11 | >98% | B |  |
| 3 | 18_CLL_V4-34_UM_tris_t14q_C.xlsx | 0 | 0 | 0 | 73 | 82 | 0 | 0 | 0 | 19568 | 4-34 | >98% | C |  |
| 3 | 13_CLL_V3-7_M_tris12_C.xlsx | 0 | 0 | 0 | 78 | 0 | 0 | 0 | 0 | 9093 | 3-7 | <98% | C |  |
| 3 | 14_CLL_V3-7_M_tris12_C.xlsx | 0 | 0 | 0 | 85 | 0 | 0 | 0 | 0 | 15257 | 3-7 | <98% | C |  |
| 3 | 15bi_CLL_V3-30_UM_tris12_del11q_B.xlsx | 0 | 0 | 0 | 93 | 0 | 91 | 0 | 0 | 5412 | 3-30 | >98% | B |  |
| 3 | 20_CLL_V3-48_M_tris12_t14q_B.xlsx | 0 | 0 | 0 | 82 | 91 | 0 | 0 | 0 | 10807 | 3-48 | <98% | B |  |
| 3 | 12_CLL_V4-39_UM_tris12_del17p_B.xlsx | 0 | 0 | 0 | 80 | 0 | 0 | 0 | 35 | 22028 | 4-39 | >98% | B |  |
| 3 | 9_CLL_V5-51_UM_tris12_B.xlsx | 0 | 0 | 0 | 76 | 0 | 0 | 0 | 0 | 175686 | 5-51 | >98% | B |  |

*The unmutated status of IGHV genes (U) was defined as those with >98% identity with the most similar germline gene, while mutated one (M) was those in which CLL-like B cell clones displayed IGHV genes with <98% identity with the most similar germline gene

**only applicable to CLL cases
