# Supplementary material for: Combined Patterns of IGHV Repertoire and Cytogenetic/Molecular Alterations in Monoclonal B Lymphocytosis versus Chronic Lymphocytic Leukemia
Source: PLoS One. 2013 Jul 3;8(7):e67751. doi: 10.1371/journal.pone.0067751 (PMC3701012; doi:10.1371/journal.pone.0067751)
Supplement: Table S3 — Heavy chain variable region (IGHV) sequences of CLL-like and CLL B-cell clones analyzed by the IMGT-V-QUEST tool. (DOCX) [file pone.0067751.s003.docx]

**Table S3.** **Heavy chain variable region (IGHV) sequences of CLL-like and CLL B-cell clones analyzed by the IMGT-V-QUEST tool**

| **B-cell clon from #case number** | **V-GENE and allele** | **Functionality** | **% Identity**  **V-REGION** | **J-GENE and allele** | **D-GENE and allele** | **D-REGION reading frame** | **CDR3 length** | **AA JUNCTION** |
| --- | --- | --- | --- | --- | --- | --- | --- | --- |
| MBLlo from (monoclonal) #1 | IGHV4-34*01 | Productive | 91.84% | IGHJ4*02 | IGHD2-15*01 | 2 | 20 | CARGPPYCSGDSCSWGGILDYW |
| MBLlo from (monoclonal) #2 | IGHV3-23*01 | Productive | 100.00% | IGHJ6*02 | IGHD3-10*01 | 1 | 11 | CANRGETRGMDVW |
| MBLlo from (monoclonal) #3 | IGHV3-7*01 | Productive | 96.39% | IGHJ4*02 | IGHD6-19*01 | 1 | 12 | CVRDKYDSGSMDYW |
| MBLlo from (monoclonal) #4 | IGHV3-21*01 | Productive | 92.34% | IGHJ6*02 | IGHD6-25*01 | 1 | 20 | CARHHPVRESSATGHYYGMDVW |
| MBLlo from (monoclonal) #5 | IGHV3-23*01 | Productive | 98.42% | IGHJ6*02 | IGHD5-12*01 | 1 | 11 | CANRGETRGMDVW |
| MBLlo from (monoclonal) #6 | IGHV3-48*03 | Productive | 90.36% | IGHJ3*02 | IGHD5-12*01 | 3 | 12 | CVRDGFHYYGFDIW |
| MBLlo from (monoclonal) #7 | IGHV3-23*01 | Productive | 95.98% | IGHJ4*02 | IGHD2-15*01 | 2 | 15 | CAKHGSYSPDPYYFDYW |
| MBLlo from (monoclonal) #8 | IGHV1-8*01 | Productive | 97.86% | IGHJ4*02 | IGHD2-21*02 | 2 | 13 | CARGLGSASQSRDSW |
| MBLlo from (monoclonal) #9 | IGHV4-34*01 | Productive | 96.57% | IGHJ4*02 | IGHD4-11*01 | 2 | 13 | CARLGGDDSDYGFYW |
| MBLlo from (monoclonal) #10 | IGHV3-7*03 | Productive | 85.54% | IGHJ4*03 | IGHD3-3*01 | 2 | 15 | CVRENEFWSGGWGLDGW |
| MBLlo from (monoclonal) #11 | IGHV3-23*01 | Productive | 99.6% | IGHJ6*02 | IGHD5-12*01 | 1 | 11 | CANRGETRGMDVW |
| MBLlo from (monoclonal) #12 | IGHV3-30*02 | Productive | 88.54% | IGHJ3*02 | IGHD1-26*01 | 3 | 20 | CANLGESRGGGSYPAPDTFDIW |
| MBLlo from (monoclonal) #13 | IGHV1-2*02 | Productive | 100.00% | IGHJ4*02 | IGHD6-19*01 | 3 | 13 | CARLQWLGISHFDYW |
| MBLlo from (multiclonal) #14A | IGHV3-23*01 | Productive | 99.6% | IGHJ6*02 | IGHD5-12*01 | 1 | 11 | CANRGETRGMDVW |
| MBLlo from (multiclonal) #14B | IGHV3-48*03 | Productive | 90.76% | IGHJ3*02 | IGHD5-12*01 | 3 | 12 | CVRDGFHYYGFDIW |
| MBLlo from (multiclonal) #15B | IGHV3-23*01 | Productive | 97.99% | IGHJ6*02 | IGHD6-19*01 | 1 | 22 | CANAPTPYSSGWNPWDYYYGMDVW |
| MBLlo from (multiclonal) #16B | IGHV3-11*03 | Productive | 96.79% | IGHJ4*02 | IGHD2-15*01 | 2 | 17 | CAREEYCDGGTCYRLFDYW |
| MBLlo from (multiclonal) #17A | IGHV3-74*01 | Productive | 90.32% | IGHJ4*02 | IGHD3-10*01 | 2 | 14 | CARDLDGSGSGVFDWW |
| MBLlo from (multiclonal) #17B | IGHV4-59*03 | Productive | 91.8% | IGHJ6*01 | IGHD3-10*01 | 2 | 15 | CARGWRSTDSYYGMDVW |
| MBLlo from (multiclonal) #18B | IGHV5-a*01 | Productive | 100.00% | IGHJ6*03 | IGHD6-19*01 | 2 | 21 | CARHVAVAGTTWGPYYYYYMDVW |
| MBLlo from (multiclonal) #19B | IGHV3-23*01 | Productive | 100.00% | IGHJ4*02 | IGHD2-2*01 | 2 | 15 | CAKDHGEQFIGGCFDYW |
| MBLlo from (multiclonal) #20B | IGHV1-69*01 | Productive | 100.00% | IGHJ3*02 | IGHD3-3*01 | 2 | 22 | CARDNPKYYDFWSGYYAPPAFDIW |
| MBLlo from (multiclonal) #21C | IGHV1-69*13 | Productive | 94.84% | IGHJ4*02 | IGHD4-11*01 | 2 | 15 | CAREGKSRDNSNPFDYW |
| MBLlo from (multiclonal) #22B | IGHV3-21*04 | Productive | 94.98% | IGHJ6*02 | IGHD2-15*01 | 3 | 9 | CARDANGMDVW |
| MBLlo from (multiclonal) #23B | IGHV4-34*01 | Productive | 96.68% | IGHJ4*02 | IGHD7-27*01 | 1 | 6 | CAHLSGYW |
| MBLlo from (multiclonal) #23C | IGHV3-11*04 | Productive | 97.57% | IGHJ6*03 | IGHD2-21*01 | 2 | 18 | CARKTCASITNYYYYYMDVW |
| MBLlo from (multiclonal) #24B | IGHV3-21*01 | Productive | 99.6% | IGHJ4*02 | IGHD6-13*01 | 2 | 11 | CARVGAATGMDYW |
| MBLhigh from (monoclonal) #25 | IGHV3-23*01 | Productive | 94.76% | IGHJ3*02 | IGHD1-7*01 | 3 | 18 | CAKDLPSTYNWNSGGAFDIW |
| MBLhigh from (monoclonal) #26 | IGHV3-23*01 | Productive | 90.36% | IGHJ4*02 | IGHD5-12*01 | 3 | 16 | CTKDPRDTGYGGDAFDYW |
| MBLhigh from (monoclonal) #27 | IGHV3-23*01 | Productive | 99.58% | IGHJ6*02 | IGHD3-3*01 | 2 | 22 | CAKDNKYYDFWSGYYPVGTGMDVW |
| MBLhigh from (monoclonal) #28 | IGHV3-53*01 | Productive | 92.31% | IGHJ3*02 | IGHD3-10*01 | 3 | 16 | CARGPPQSRPVGDTFEIW |
| MBLhigh from (monoclonal) #29 | IGHV2-26*01 | Productive | 91.64% | IGHJ4*03 | IGHD5-18*01 | 3 | 15 | CTRTRGYPYGDRYFDSW |
| MBLhigh from (monoclonal) #30 | IGHV1-2*02 | Productive | 94.44% | IGHJ3*02 | IGHD4-17*01 | 2 | 13 | CARGLNTDYGAFDIW |
| MBLhigh from (monoclonal) #31 | IGHV1-2*02 | Productive | 91.88% | IGHJ3*02 | IGHD2-21*02 | 3 | 18 | CARDRSVIVVTYILDAFDMW |
| MBLhigh from (monoclonal) #32 | IGHV3-23*01 | Productive | 91.97% | IGHJ5*01 | IGHD6-13*01 | 1 | 8 | CSKGGWGDSW |
| MBLhigh from (monoclonal) #33 | IGHV3-74*01 | Productive | 94.51% | IGHJ5*02 | IGHD2-8*02 | 3 | 9 | CARQLDMYSLW |
| MBLhigh from (monoclonal) #34 | IGHV4-34*01 | Productive | 92.68% | IGHJ6*02 | IGHD3-16*01 | 2 | 20 | CVRGYPSDYTERRYYYYGLDVW |
| MBLhigh from (monoclonal) #35 | IGHV3-30*04 | Productive | 90.76% | IGHJ3*02 | IGHD2-2*01 | 2 | 18 | CTRPHCSMSSCSWNDAFAIW |
| MBLhigh from (monoclonal) #36 | IGHV3-23*01 | Productive | 91.94% | IGHJ3*02 | IGHD3-22*01 | 2 | 14 | CAKFYDDIQPNAFDIW |
| MBLhigh from (monoclonal) #37 | IGHV1-2*02 | Productive | 91.7% | IGHJ4*02 | IGHD5-18*01 | 3 | 15 | CARDLEMRYSQGSFDSW |
| MBLhigh from (monoclonal) #38 | IGHV3-11*01 | Productive | 99.2% | IGHJ6*02 | IGHD3-3*01 | 2 | 23 | CARDRRDDFWSGYRIYYYYYGMDVW |
| MBLhigh from (monoclonal) #39 | IGHV3-21*01 | Productive | 97.88% | IGHJ6*02 | IGHD1-26*01 | 3 | 9 | CARDANGMDVW |
| MBLhigh from (monoclonal) #40 | IGHV3-7*03 | Productive | 97.19% | IGHJ4*02 | IGHD6-19*01 | 1 | 9 | CARGGWYGDYW |
| MBLhigh from (monoclonal) #41 | IGHV4-39*01 | Productive | 100.00% | IGHJ6*02 | IGHD2-2*01 | 2 | 22 | CARHRLGYCSSTSCYYYYYGMDVW |
| MBLhigh from (monoclonal) #42 | IGHV3-15*01 | Productive | 91.39% | IGHJ4*02 | IGHD2-8*01 | 3 | 12 | CTTDSMVYVDMDYW |
| MBLhigh from (monoclonal) #43 | IGHV3-48*02 | Productive | 100.00% | IGHJ6*02 | IGHD2-2*01 | 3 | 23 | CARDNTANDIVVVPADYYYYGMDVW |
| MBLhigh from (monoclonal) #44 | IGHV3-23*01 | Productive | 90.76% | IGHJ3*02 | IGHD4-17*01 | 3 | 17 | CAKDRTLATVIQKDTFDIW |
| MBLhigh from (monoclonal) #45 | IGHV5-51*01 | Productive | 100.00% | IGHJ6*02 | IGHD3-3*01 | 2 | 19 | CARRDFRGDFWSGYYYGMDVW |
| MBLhigh from (monoclonal) #46 | IGHV3-23*01 | Productive | 95.32% | IGHJ6*02 | IGHD2-2*01 | 2 | 14 | CAFHCCRISCYGVDFW |
| MBLhigh from (monoclonal) #47 | IGHV4-34*01 | Productive | 93.67% | IGHJ4*01 | IGHD1-1*01 | 3 | 14 | CARVIGDKGGYYLTYW |
| MBLhigh from (monoclonal) #48 | IGHV3-48*01 | Productive | 99.59% | IGHJ6*02 | IGHD3-3*01 | 2 | 20 | CARSPGYDFWSGYPDYYGMDVW |
| MBLhigh from (monoclonal) #49 | IGHV1-2*02 | Productive | 96.02% | IGHJ4*02 | IGHD3-22*01 | 2 | 20 | CARDLARYDSGGSYKRKMFDYW |
| MBLhigh from (multiclonal) #50B | IGHV3-11*03 | Productive | 95.32% | IGHJ4*02 | IGHD6-19*01 | 1 | 10 | CAKVRSHYFDYW |
| MBLhigh from (multiclonal) #51B | IGHV3-21*04 | Productive | 96.65% | IGHJ6*02 | IGHD2-15*01 | 3 | 9 | CARDANGMDVW |
| MBLhigh from (multiclonal) #52B | IGHV3-33*01 | Productive | 99.6% | IGHJ4*02 | IGHD3-9*01 | 1 | 21 | CARDPRVLRYFDWLLSPPPFDYW |
| MBLhigh from (multiclonal) #53A | IGHV3-15*01 | Productive | 89.03% | IGHJ4*02 | IGHD2-21*01 | 2 | 12 | CTTESGWYSASDHW |
| MBLhigh from (multiclonal) #53B | IGHV3-30*03 | Productive | 94.27% | IGHJ4*02 | IGHD2-15*01 | 2 | 18 | CAKDTWGHCSGGFCSHFDSW |
| MBLhigh from (multiclonal) #54B | IGHV3-48*03 | Productive | 90.95% | IGHJ3*02 | IGHD3-3*01 | 2 | 20 | CVRDDRSCSSNNCHALRSFDMW |
| MBLhigh from (multiclonal) #55B | IGHV3-53*01 | Productive | 92.55% | IGHJ6*02 | IGHD2-8*01 | 1 | 19 | CATHPTNIYTRWPYVSDMDVW |
| MBLhigh from (multiclonal) #56A | IGHV3-23*01 | Productive | 100.00% | IGHJ6*02 | IGHD3-3*01 | 1 | 11 | CAKDWESWGMDVW |
| MBLhigh from (multiclonal) #57A | IGHV4-39*01 | Productive | 100.00% | IGHJ5*02 | IGHD6-13*01 | 1 | 19 | CASVQGYSSSWYGGDNWFDPW |
| MBLhigh from (multiclonal) #57B | IGHV3-23*01 | Productive | 100.00% | IGHJ6*02 | IGHD3-10*01 | 1 | 11 | CANRGETRGMDVW |
| MBLhigh from (multiclonal) #58A | IGHV4-39*01 | Productive | 100.00% | IGHJ5*02 | IGHD3-3*01 | 2 | 20 | CARHTSLYDFWSGYYRGWFDPW |
| MBLhigh from (multiclonal) #58B | IGHV1-69*01 | Productive | 100.00% | IGHJ4*02 | IGHD5-18*01 | 2 | 17 | CAREAGSIQLWPPGFFDYW |
| MBLhigh from (multiclonal) #59A | IGHV3-7*01 | Productive | 93.57% | IGHJ3*02 | IGHD5-12*01 | 2 | 9 | CARGRYVYDIW |
| MBLhigh from (multiclonal) #60A | IGHV4-34*01 | Productive | 96.48% | IGHJ4*02 | IGHD5-24*01 | 1 | 19 | CARAEGQATLLSVWEYYFDSW |
| MBLhigh from (multiclonal) #60B | IGHV3-33*01 | Productive | 96.67% | IGHJ2*01 | IGHD6-19*01 | 1 | 17 | CARDILITGGRGDWYFDLW |
| MBLhigh from (multiclonal) #61B | IGHV3-72*01 | Productive | 95.92% | IGHJ5*02 | IGHD2-2*01 | 2 | 13 | CVRSSTGWTDWFDPW |
| MBLhigh from (multiclonal) #62B | IGHV4-34*01 | Productive | 96.81% | IGHJ6*03 | IGHD3-3*01 | 2 | 16 | CARREDDNFWSGFYMDVW |
| MBLhigh from (multiclonal) #63B | IGHV3-23*01 | Productive | 95.16% | IGHJ4*02 | IGHD2-15*01 | 2 | 18 | CAKLSTPCGGGSCYSSLDYW |
| MBLhigh from (multiclonal) #64B | IGHV4-34*01 | Productive | 95.12% | IGHJ4*02 | IGHD6-19*01 | 1 | 13 | CARRDSSGWYYFDYW |
| MBLhigh from (multiclonal) #65A | IGHV5-51*01 | Productive | 92.2% | IGHJ4*01 | IGHD5-18*01 | 3 | 14 | CGRRRTGYNDGEIDYW |
| MBLhigh from (multiclonal) #65B | IGHV4-30-4*01 | Productive | 97.81% | IGHJ4*01 | IGHD2-2*01 | 2 | 16 | CARHPSCSRTSCYFFDYW |
| MBLhigh from (multiclonal) #66A | IGHV5-51*01 | Productive | 100.00% | IGHJ4*02 | IGHD3-3*01 | 2 | 21 | CARHGTYYDFWSGYYLPGFFDYW |
| MBLhigh from (multiclonal) #66B | IGHV1-69*01 | Productive | 100.00% | IGHJ6*02 | IGHD6-13*01 | 1 | 26 | CARQGAGSSWYGIVKGWFEYYYYGMDVW |
| MBLhigh from (multiclonal) #66C | IGHV3-33*01 | Productive | 98.81% | IGHJ3*02 | IGHD3-3*01 | 1 | 22 | CARGNGGALRFLEWLLYHDAFDIW |
| MBLhigh from (multiclonal) #67A | IGHV1-3*01 | Productive | 97.96% | IGHJ6*02 | IGHD3-3*01 | 2 | 23 | CARADGGYDFWSGYSTVNYYGMDVW |
| MBLhigh from (multiclonal) #67B | IGHV3-9*01 | Productive | 93.57% | IGHJ4*02 | IGHD1-26*01 | 3 | 14 | CARVESGSYFWPSDYW |
| CLL from (monoclonal) #68 | IGHV3-23*01 | Productive | 93.57% | IGHJ4*02 | IGHD4-23*01 | 1 | 12 | CAKGRQLWSYLDYW |
| CLL from (monoclonal) #69 | IGHV1-2*02 | Productive | 99.59% | IGHJ3*02 | IGHD5-12*01 | 3 | 12 | CARDGDYFDAFDIW |
| CLL from (monoclonal) #70 | IGHV3-11*01 | Productive | 98.8% | IGHJ4*02 | IGHD3-3*01 | 2 | 22 | CARDPRYYDFWSGYYLPDDKFDYW |
| CLL from (monoclonal) #71 | IGHV3-7*01 | Productive | 93.98% | IGHJ4*02 | - | - | 8 | CASGSHVDYY (TRP 118 not identified) |
| CLL from (monoclonal) #72 | IGHV4-b*02 | Productive | 100.00% | IGHJ4*02 | IGHD5-18*01 | 2 | 13 | CARSWIQLWSEFDYW |
| CLL from (monoclonal) #73 | IGHV4-4*02 | Productive | 91.57% | IGHJ6*02 | IGHD5-12*01 | 1 | 25 | CARGSRNVDIVATITFIGFYYYGMDVW |
| CLL from (monoclonal) #74 | IGHV1-69*06 | Productive | 97.96% | IGHJ6*03 | IGHD3-3*01 | 2 | 24 | CARAEQYYDFWSGHKGVDYYYYMDVW |
| CLL from (monoclonal) #75 | IGHV3-11*01 | Productive | 97.91% | IGHJ4*02 | IGHD3-10*01 | 2 | 16 | CARGPDPYYYGSGTPSYW |
| CLL from (monoclonal) #76 | IGHV3-21*01 | Productive | 97.21% | IGHJ5*02 | IGHD3-9*01 | 1 | 22 | CARDRRNGNFDWLEDPLYNWFDPW |
| CLL from (monoclonal) #77 | IGHV3-9*01 | Productive | 90.91% | IGHJ6*02 | IGHD4-23*01 | 1 | 16 | CAKDRSNTWPLWGGMDVW |
| CLL from (monoclonal) #78* | IGHV1-2*02 | Productive | 99.57% | IGHJ4*02 | IGHD6-19*01 | 3 | 13 | CARAQWLVLENFDYW |
| CLL from (monoclonal) #79 | IGHV4-34*01 | Productive | 85.43% | IGHJ3*02 | IGHD5-12*01 | 2 | 17 | CARREEDWKRSGRDSFDIW |
| CLL from (monoclonal) #80 | IGHV1-69*01 | Productive | 95.1% | IGHJ4*02 | IGHD3-3*01 | 2 | 13 | CAKGPYYDFWSGDYW |
| CLL from (monoclonal) #81 | IGHV4-39*01 | Productive | 99.21% | IGHJ4*02 | IGHD3-16*02 | 2 | 19 | CARHTYYDYVWGSYRTPFDYW |
| CLL from (monoclonal) #82 | IGHV3-21*01 | Productive | 100.00% | IGHJ4*02 | IGHD2-2*02 | 2 | 21 | CAREGGLGYCSSTSCYTTLFDYW |
| CLL from (monoclonal) #83 | IGHV3-72*01 | Productive | 89.41% | IGHJ4*02 | IGHD6-13*01 | 3 | 15 | CVRSSMGAEQTIACDYW |
| CLL from (monoclonal) #84 | IGHV3-30*01 | Productive | 95.2% | IGHJ4*02 | IGHD6-19*01 | 1 | 15 | CARDDYSSGVGTRLSYW |
| CLL from (monoclonal) #85 | IGHV4-4*03 | Productive | 92.77% | IGHJ6*02 | IGHD2-2*01 | 2 | 21 | CARAPYCGSNTCYSYYYYGMDVW |
| CLL from (monoclonal) #86 | IGHV4-34*01 | Productive | 89.72% | IGHJ6*02 | IGHD2-15*01 | 2 | 24 | CAGRFYCSGDTCHLPLYHYYYGLDVW |
| CLL from (monoclonal) #87 | IGHV3-23*01 | Productive | 94.78% | IGHJ6*04 | IGHD3-3*01 | 2 | 23 | CARDLTHHNFWSAYYETSYCGMDVW |
| CLL from (monoclonal) #88 | IGHV3-48*02 | Productive | 95.58% | IGHJ6*03 | IGHD5-24*01 | 3 | 18 | CARQGEDYNNRGYYCYMDVW |
| CLL from (monoclonal) #89 | IGHV1-2*02 | Productive | 98.29% | IGHJ6*02 | IGHD3-10*01 | 2 | 17 | CARDPGGGDYYYYYGMDVW |
| CLL from (monoclonal) #90 | IGHV4-39*01 | Productive | 92.97% | IGHJ4*02 | IGHD6-13*01 | 3 | 10 | CARHEQQLADYW |
| CLL from (monoclonal) #91 | IGHV4-34*01 | Productive | 95.98% | IGHJ6*02 | IGHD4-23*01 | 3 | 20 | CARGYGSTGETRRYYYYGMDVW |
| CLL from (monoclonal) #92 | IGHV4-59*01 | Productive | 100.00% | IGHJ4*02 | IGHD3-3*01 | 2 | 19 | CARVVHYLDFWSGYTYYFDYW |
| CLL from (monoclonal) #93 | IGHV3-64*01 | Productive | 95.58% | IGHJ6*02 | IGHD6-19*01 | 2 | 9 | CAVDRTGMDVW |
| CLL from (monoclonal) #94 | IGHV6-1*01 | Productive | 92.19% | IGHJ4*02 | IGHD6-19*01 | 1 | 18 | CARSPSRYSNGWYERDFDCW |
| CLL from (monoclonal) #95 | IGHV1-69*01 | Productive | 100.00% | IGHJ6*02 | IGHD6-19*01 | 2 | 21 | CAREVVYGVAGTYYYYYYGMDVW |
| CLL from (monoclonal) #96 | IGHV4-34*08 | Productive | 85.96% | IGHJ4*02 | IGHD3-22*01 | 2 | 13 | CARGFHWGGYYLDFW |
| CLL from (monoclonal) #97 | IGHV3-23*01 | Productive | 94.4% | IGHJ4*02 | IGHD2-15*01 | 2 | 18 | CAKLSTPCGGGSCYSSLDYW |
| CLL from (monoclonal) #98 | IGHV1-8*01 | Productive | 87.5% | IGHJ6*02 | IGHD3-3*01 | 2 | 21 | CARGPSYYDFWSGPFDNYGMDVW |
| CLL from (monoclonal) #99* | IGHV1-2*02 | Productive | 100.00% | IGHJ4*02 | IGHD6-19*01 | 3 | 13 | CARAQWLVLENFDYW |
| CLL from (monoclonal) #100 | IGHV3-7*01 | Productive | 94.38% | IGHJ3*01 | IGHD3-16*01 | 1 | 16 | CASALRYLPYADTAFDLW |
| CLL from (monoclonal) #101 | IGHV3-7*03 | Productive | 85.77% | IGHJ4*03 | IGHD3-3*01 | 2 | 15 | CVRENEFWSGGWGLDGW |
| CLL from (monoclonal) #102 | IGHV1-69*13 | Productive | 99.59% | IGHJ6*02 | IGHD3-3*01 | 3 | 22 | CATTTITIFGVVTVYYYYYGMDVW |
| CLL from (monoclonal) #103 | IGHV4-34*01 | Productive | 100.00% | IGHJ4*02 | IGHD3-10*01 | 2 | 20 | CARGLIGAYGSGSYYPFPFDYW |
| CLL from (monoclonal) #104 | IGHV1-69*01 | Productive | 100.00% | IGHJ6*02 | IGHD3-3*01 | 2 | 23 | CARADGGYDFWSGYSTVNYYGMDVW |
| CLL from (monoclonal) #105 | IGHV3-48*03 | Productive | 91.06% | IGHJ3*02 | IGHD5-12*01 | 3 | 12 | CVRDGFHYYGFDIW |
| CLL from (monoclonal) #106 | IGHV3-30*03 | Productive | 97.93% | IGHJ6*03 | IGHD3-3*01 | 2 | 30 | CAKDQEQGPRPRYYDFWSAPPPWYYYYYMDVW |
| CLL from (monoclonal) #107 | IGHV1-69*01 | Productive | 98.29% | IGHJ5*02 | IGHD3-3*01 | 2 | 15 | CATDKKYYDFWSGYYLW |
| CLL from (monoclonal) #108 | IGHV4-59*02 | Productive | 91.5% | IGHJ4*02 | IGHD1-14*01 | 3 | 13 | CARHLRNDKYYLDFW |
| CLL from (monoclonal) #109 | IGHV3-48*03 | Productive | 100.00% | IGHJ6*02 | IGHD3-3*01 | 2 | 21 | CARDYDFWSGYYSYYYYYGMDVW |
| CLL from (monoclonal) #110 | IGHV5-51*01 | Productive | 99.6% | IGHJ6*03 | IGHD2-2*01 | 2 | 23 | CARYCSSTSCMTGTMGYYYYYMDVW |
| CLL from (monoclonal) #111 | IGHV3-48*02 | Productive | 92.34% | IGHJ4*02 | IGHD6-13*01 | 1 | 14 | CARDLGGNSWPTFDFW |
| CLL from (monoclonal) #112 | IGHV3-30*03 | Productive | 89.56% | IGHJ6*02 | IGHD6-19*01 | 2 | 19 | CAKIGMAGDFLEFRYYGMDVW |
| CLL from (monoclonal) #113 | IGHV1-3*01 | Productive | 99.18% | IGHJ6*02 | IGHD3-22*01 | 2 | 25 | CARDLTYYYDSSGYYYFNYYYYGMDVW |
| CLL from (monoclonal) #114 | IGHV3-7*03 | Productive | 85.54% | IGHJ4*03 | IGHD3-3*01 | 2 | 15 | CVRENEFWSGGWGLDGW |
| CLL from (monoclonal) #115 | IGHV4-59*01 | Productive | 91.94% | IGHJ4*02 | IGHD6-19*01 | 1 | 14 | CARGPDISGWNGLDYW |
| CLL from (monoclonal) #116 | IGHV4-34*01 | Productive | 91.46% | IGHJ5*02 | IGHD6-13*01 | 3 | 13 | CATNSRESQGWFDPW |
| CLL from (monoclonal) #117 | IGHV3-15*01 | Productive | 91.32% | IGHJ6*02 | IGHD2-15*01 | 2 | 23 | CVTGPGYCSGGGCSSRGYYYGMDVW |
| CLL from (monoclonal) #118 | IGHV3-30*04 | Productive | 100.00% | IGHJ6*02 | IGHD3-3*01 | 2 | 22 | CARDLKTAYYDFWSGYYGDGMDVW |
| CLL from (monoclonal) #119 | IGHV3-9*01 | Productive | 98.8% | IGHJ6*02 | IGHD3-3*01 | 2 | 26 | CAKDKYYDFWSGYSHLGVLYYYYGMDVW |
| CLL from (monoclonal) #120 | IGHV1-3*01 | Productive | 97.94% | IGHJ6*02 | IGHD3-3*01 | 2 | 23 | CARADGGYDFWSGYSTVNYYGMDVW |
| CLL from (monoclonal) #121 | IGHV1-18*01 | Productive | 100.00% | IGHJ6*02 | IGHD2-15*01 | 3 | 9 | CARDANGMDVW |
| CLL from (monoclonal) #122 | IGHV1-69*01 | Productive | 100.00% | IGHJ3*02 | IGHD3-16*02 | 2 | 21 | CARGGNYDYIWGSYRPNDAFDIW |
| CLL from (monoclonal) #123 | IGHV1-69*02 | Productive | 100.00% | IGHJ6*03 | IGHD2-15*01 | 3 | 22 | CARSQAHIVVVVAATYYYYYMDVW |
| CLL from (monoclonal) #124 | IGHV1-18*01 | Productive | 100.00% | IGHJ4*02 | IGHD7-27*01 | 3 | 13 | CARKNWGPDYYFDYW |
| CLL from (monoclonal) #125 | IGHV4-39*01 | Productive | 100.00% | IGHJ6*01 | IGHD2-2*01 | 2 | 22 | CARHRLGYCSSTSCYYYYYGMDVW |
| CLL from (monoclonal) #126 | IGHV1-69*01 | Productive | 98.76% | IGHJ1*01 | IGHD3-22*01 | 2 | 22 | CARGSSTYYYDSSVYGVAEYFQHW |
| CLL from (monoclonal) #127 | IGHV3-30*03 | Productive | 98.39% | IGHJ4*02 | IGHD3-22*01 | 2 | 21 | CARGPNVSHTYYYDNSGSHFDYW |
| CLL from (monoclonal) #128 | IGHV3-74*01 | Productive | 92.98% | IGHJ4*02 | IGHD2-2*01 | 3 | 15 | CARVDIEVDGGGHFDNW |
| CLL from (monoclonal) #129 | IGHV1-2*02 | Productive | 96.97% | IGHJ4*02 | IGHD2-8*01 | 1 | 15 | CGRDVELRYWQGYFDLW |
| CLL from (monoclonal) #130 | IGHV1-18*01 | Productive | 100.00% | IGHJ6*02 | IGHD6-13*01 | 1 | 19 | CARDLSLSSNWFTPPYGMDVW |
| CLL from (monoclonal) #131 | IGHV1-2*02 | Productive | 100.00% | IGHJ5*01 | IGHD3-3*01 | 2 | 32 | CARAPRGDYDTEAGGAYSYGLEVWRLRRNRFDSW |
| CLL from (monoclonal) #132 | IGHV4-39*07 | Productive | 100.00% | IGHJ6*02 | IGHD2-2*01 | 2 | 22 | CARDRLGYCSSTSCYLYYYGMDVW |
| CLL from (monoclonal) #133 | IGHV4-b*01 | Productive | 94.14% | IGHJ1*01 | IGHD2-15*01 | 2 | 20 | CARLPHCTASRCYGGGRYVDQW |
| CLL from (monoclonal) #134 | IGHV1-18*01 | Productive | 99.57% | IGHJ6*02 | IGHD3-9*01 | 2 | 20 | CARGAYYDILTGYRYYYGMDVW |
| CLL from (monoclonal) #135 | IGHV4-34*01 | Productive | 95.49% | IGHJ4*02 | IGHD2-15*01 | 2 | 11 | CARGSAGSRLDYW |
| CLL from (monoclonal) #136 | IGHV3-21*01 | Productive | 94.63% | IGHJ6*02 | - | - | 9 | RTKDANGMDVW (2nd-CYS 104 not identified) |
| CLL from (monoclonal) #137 | IGHV3-7*01 | Productive | 95.98% | IGHJ4*02 | IGHD2-2*01 | 2 | 16 | CGSQCSTTSCPSSISEYW |
| CLL from (monoclonal) #138 | IGHV1-69*01 | Productive | 100.00% | IGHJ6*03 | IGHD7-27*01 | 3 | 21 | CARDTGLMTNWGYYYYYYYMDVW |
| CLL from (monoclonal) #139 | IGHV3-7*03 | Productive | 92.59% | IGHJ4*02 | IGHD3-22*01 | 2 | 15 | CARVSDETTGYGNFDYW |
| CLL from (monoclonal) #140 | IGHV4-b*01 | Productive | 99.59% | IGHJ4*02 | IGHD5-18*01 | 2 | 13 | CARAWIQLWSDFDYW |
| CLL from (monoclonal) #141 | IGHV4-34*01 | Productive | 99.19% | IGHJ6*02 | IGHD2-2*01 | 3 | 21 | CARADLLVVPAAIYYYYYGMDVW |
| CLL from (monoclonal) #142 | IGHV3-48*03 | Productive | 91.13% | IGHJ6*02 | IGHD6-19*01 | 1 | 8 | CSRRGRLDIW |
| CLL from (monoclonal) #143 | IGHV3-30*03 | Productive | 96.37% | IGHJ4*02 | IGHD3-10*01 | 2 | 19 | CANRGDTSGLGTCCQGIGDSW |
| CLL from (monoclonal) #144 | IGHV4-61*02 | Productive | 91.63% | IGHJ5*02 | IGHD5-12*01 | 3 | 14 | CAKRYGDHGEGWFDPW |
| CLL from (monoclonal) #145 | IGHV3-30*01 | Productive | 93.15% | IGHJ4*02 | IGHD3-10*01 | 3 | 17 | CASGSMIGGVILPPGFDYW |
| CLL from (monoclonal) #146 | IGHV3-53*01 | Productive | 100.00% | IGHJ6*02 | IGHD3-22*01 | 2 | 25 | CAREGYYDSSGYSEAPHYYYYYGMDVW |
| CLL from (monoclonal) #147 | IGHV2-70*11 | Productive | 96.21% | IGHJ4*02 | IGHD1-20*01 | 3 | 14 | CARMQHRYHWNDSDSW |
| CLL from (monoclonal) #148 | IGHV1-3*01 | Productive | 90.2% | IGHJ5*01 | IGHD3-9*01 | 1 | 19 | CARGIRYSGWLLYGSDWYDSW |
| CLL from (monoclonal) #149 | IGHV3-23*01 | Productive | 99.58% | IGHJ6*02 | IGHD5-12*01 | 1 | 11 | CANRGETRGMDVW |
| CLL from (monoclonal) #150 | IGHV4-39*01 | Productive | 100.00% | IGHJ5*02 | IGHD6-13*01 | 1 | 18 | CATQTGYSSSWYAVNWFDPW |
| CLL from (monoclonal) #151 | IGHV1-24*01 | Productive | 100.00% | IGHJ6*02 | IGHD3-9*01 | 2 | 24 | CATDGYDILTGYYKGPGAYYYGMDVW |
| CLL from (monoclonal) #152 | IGHV3-48*03 | Productive | 99.16% | IGHJ3*02 | IGHD5-12*01 | 3 | 12 | CARDGFHYYGFDIW |
| CLL from (monoclonal) #153 | IGHV4-61*02 | Productive | 92.65% | IGHJ4*02 | IGHD7-27*01 | 3 | 12 | CARDNWGFEGFDSW |
| CLL from (monoclonal) #154 | IGHV3-74*01 | Productive | 93.15% | IGHJ3*01 | IGHD4-23*01 | 3 | 14 | CARGHKVVNPGSFDLW |
| CLL from (multiclonal) #155A | IGHV4-34*01 | Productive | 93.06% | IGHJ4*02 | IGHD3-3*01 | 3 | 11 | CARPNVGAVFVFW |
| CLL from (multiclonal) #156A | IGHV4-34*01 | Productive | 96.69% | IGHJ2*01 | IGHD4-23*01 | 2 | 13 | CARAGGYSDWYFDLW |
| CLL from (multiclonal) #157A | IGHV3-9*01 | Productive | 100.00% | IGHJ3*02 | IGHD3-3*01 | 2 | 19 | CAKDRYYDFWSGYYTAAFDIW |
| CLL from (multiclonal) #158A | IGHV4-39*01 | Productive | 98.47% | IGHJ6*02 | IGHD3-3*01 | 1 | 18 | CGILGEWLSFYFFFYGMDVW |
| CLL from (multiclonal) #159A | IGHV3-30-3*01 | Productive | 91.13% | IGHJ4*02 | IGHD5-12*01 | 3 | 16 | CARGKGRNSGYDYLLHYW |
| CLL from (multiclonal) #160A | IGHV1-3*01 | Productive | 94.03% | IGHJ5*02 | IGHD5-18*01 | 1 | 19 | CARDRVVIIPDTTTINWFDPW |
| CLL from (multiclonal) #161A | IGHV3-48*02 | Productive | 96.34% | IGHJ4*02 | IGHD4-17*01 | 2 | 12 | CARSSGDDSLIDYW |
| CLL from (multiclonal) #162A | IGHV4-34*01 | Productive | 97.56% | IGHJ6*02 | IGHD3-10*01 | 2 | 17 | CARGFDYYGSGSANGLDVW |
| CLL from (multiclonal) #163A | IGHV1-46*01 | Productive | 100.00% | IGHJ4*01 | IGHD3-3*01 | 2 | 21 | CARAHYYDFWSGYVYPRLAFDYW |
| CLL from (multiclonal) #164A | IGHV3-53*01 | Productive | 100.00% | IGHJ6*02 | IGHD3-22*01 | 2 | 25 | CAREGYYDSSGYSEAPHYYYYYGMDVW |
| CLL from (multiclonal) #165A | IGHV1-3*01 | Productive | 96.2% | IGHJ3*01 | IGHD1-26*01 | 3 | 17 | CARGLRSGTFYGADAFDFW |
| CLL from (multiclonal) #165B | IGHV4-34*01 | Productive | 92.93% | IGHJ3*02 | IGHD2-15*01 | 3 | 26 | TARGGLFVETEIAGVGYRSGTGTLFDSW  (2nd-CYS 104 not identified) |
| CLL from (multiclonal) #166A | IGHV3-33*01 | Productive | 91.5% | IGHJ6*02 | IGHD3-10*01 | 2 | 18 | CARDDNRDGSGNYKGGMDFW |
| CLL from (multiclonal) #166B | IGHV3-21*01 | Productive | 94.8% | IGHJ4*02 | IGHD4-23*01 | 2 | 13 | CARDLDGGNSVFDYW |
| CLL from (multiclonal) #167A | IGHV3-52*01(P) | Productive** | 96.06% | IGHJ3*02 | IGHD2-21*02 | 2 | 19 | CMTVLWANRGGDCPGDAFDIW |
| CLL from (multiclonal) #168A | IGHV4-34*01 | Productive | 98.78% | IGHJ4*02 | IGHD1-26*01 | 3 | 17 | CARGPDRLYSGSYTRFDYW |
| CLL from (multiclonal) #169A | IGHV3-48*02 | Productive | 93.6% | IGHJ4*02 | IGHD3-10*01 | 1 | 12 | CVRELWFGNGGDYW |
| CLL from (multiclonal) #170A | IGHV3-30*03 | Productive | 99.22% | IGHJ6*02 | IGHD3-9*01 | 1 | 25 | CAKYGGVKLRYFDWLLYGDYYYGMDVW |
| CLL from (multiclonal) #171A | IGHV3-33*01 | Productive | 99.61% | IGHJ5*02 | IGHD1-26*01 | 2 | 12 | CARGELLHNWFDPW |
| CLL from (multiclonal) #171B | IGHV3-23*01 | Productive | 99.18% | IGHJ3*02 | IGHD5-12*01 | 3 | 12 | CAKDGFPYYGFDIW |
| CLL from (multiclonal) #172A | IGHV2-26*01 | Productive | 91.91% | IGHJ5*02 | IGHD3-3*01 | 2 | 22 | CAGTNIPRQFDFWSGSSPNWFDPW |
| CLL from (multiclonal) #173A | IGHV3-30*03 | Productive | 99.6% | IGHJ6*02 | IGHD3-9*01 | 1 | 25 | CAKYGGVKLRYFDWLLYGDYYYGMDVW |
| CLL from (multiclonal) #174A | IGHV4-39*01 | Productive | 98.76% | IGHJ3*01 | IGHD1-14*01 | 3 | 17 | CASHRNTQTYNNRAAFDVW |
| CLL from (multiclonal) #175A | IGHV5-51*01 | Productive | 100.00% | IGHJ4*02 | IGHD2-15*01 | 2 | 19 | CARIPVAGYCRGGSCYPFDYW |
| CLL from (multiclonal) #175B | IGHV4-34*01 | Productive | 99.16% | IGHJ4*02 | IGHD3-10*01 | 2 | 14 | CARTKTYGSGPPGKYW |
| CLL from (multiclonal) #176A | IGHV4-34*01 | Productive | 99.59% | IGHJ4*02 | IGHD3-10*01 | 2 | 15 | CARGLYYYGSGVYFDYW |

*B-cell clones from #78 and #99 corresponded to the same untreated CLL patient at recruitment and after 1-year evaluation, respectively

**however the closest V is a pseudogene

Grey shadowed cells highlight those complementary-determining regions (CDR3) of the IGHV genes identical or highly homologous

Scripts in cells indicate IMGT/JunctionAnalysis giving no results for that JUNCTION
