# Supplementary material for: Combined Patterns of IGHV Repertoire and Cytogenetic/Molecular Alterations in Monoclonal B Lymphocytosis versus Chronic Lymphocytic Leukemia
Source: PLoS One. 2013 Jul 3;8(7):e67751. doi: 10.1371/journal.pone.0067751 (PMC3701012; doi:10.1371/journal.pone.0067751)
Supplement: Materials and Methods S1 — (DOCX) [file pone.0067751.s004.docx]

SUPPLEMENTAL MATERIAL & METHODS

#### Immunophenotypic analysis. EDTA-anticoagulated peripheral blood (PB) samples were immunophenotyped using a direct immunofluorescence stain-and-then-lyse technique, with the following multicolor antibody stainings: 1) CD20-pacific blue (PacB)/ CD45-pacific orange (PacO)/ CD8-fluorescein isothiocyanate (FITC)+anti-surface immunoglobulin (sIg)λ-FITC / CD56-phycoerythrin (PE) +anti-sIgκ-PE/ CD4-peridinin chlorophyll protein-cyanin 5.5 (PerCPCy5.5)/ CD19-PE–cyanin 7 (PE-Cy7)/ CD3-allophycocyanin (APC)/ CD38-AlexaFluor 700 (AF700); 2) CD20-PacB/ CD45-PacO/ cytoplasmic (Cy)bcl2-FITC/ CD23-PE/ CD19-PerCPCy5.5/ CD10/-PE-Cy7 CD5-APC/ CD38-AF700 and 3) CD20-PB/ anti-sIgλ-FITC/ anti-sIgκ-PE/ CD19-PerCPCy5.5/ CD10-PE-Cy7/ CD5-APC. As all cases included in this study showed clonal and/or aberrant B-cell population(s), the study was extended with the following additional 5- and 6-color stainings (PacB/FITC/PE/PerCPCy5.5/PECy7/APC): 1) CD20/ CD22/ CCR6/ -/ CD19/ CD5; 2) CD20/ CD103/ CD25/ CD5/ CD19/ CD11c; 3) CD20/ CD43/ CD79b/ CD5/ CD19/ CD49d; 4) CD20/ sIgM/ CD27/ -/ CD19/ CD5; 5) CD20/ FMC7/ CD24/ - /CD19/CD5; and 6) CD20/ CD3/ Cyzap70/ -/ CD19/ CD5. For the staining of Cybcl2 and Cyzap70, the Fix & Perm™ reagent kit (Invitrogen, Carlsbad, CA, USA) was used, following the recommendations of the manufacturer. All reagents were purchased from Becton/Dickinson Biosciences (BDB; San Jose, CA, USA), except for CD19-PECy7 (Beckman/Coulter, Miami, FL), CD20-PacB (e-Biosciences, San Diego, CA, USA), CD38-AF700 (Exbio, Prague, Czech Republic), CD45-PacO (Invitrogen, Carlsbad, CA, USA), CD79bPE, CD24PE and CD43FITC (Immunotech, Marseille, France), and bcl2-FITC, anti-IgM-FITC, anti-sIgλ-FITC and anti-sIgκ-PE (DAKO, Glostrup, Denmark).

Data acquisition was performed on a FACSCanto II flow cytometer (BDB) using the FACSDiva software (v6.1; BDB); in MBL^lo^ cases, acquisition was done using a double-step procedure: firstly, information on 1x10^5^ events corresponding to the whole sample cellularity was stored; in the second step, information was stored on CD19+ and/or CD20+ gated events, containing a minimum of 5x10^6^ leucocytes/tube. Instrument setup and calibration were performed according to well-established protocols, and a daily quality control program was followed, using the *Cytometer Setup and Tracking (CST)*™  *Beads* and *CST Module* (BDB). Data analysis was performed using the INFINICITY^TM^ software program (Cytognos SL, Salamanca, Spain). B lymphocytes were identified according to their SSC/CD19^+^ distribution and their numbers were calculated after excluding cell debris and platelets. The definition of a CLL-like/CLL phenotype was based on the presence of a CD19^+^, CD5^+^, CD20^lo^, CD23^+^, CD79b^lo^, FMC7^-/lo^, Cybcl2^hi^ and sIgκ^lo^ or sIgλ^lo^, in the absence of CD10 expression. The minimum number of cellular events required to define the presence of a CLL-like / CLL B-cell cluster was of 50 cells.

The frequency of distribution of sIgκ^+^ *vs*. sIgλ^+^ populations within the CLL-like/CLL B cells were visually analyzed by superimposing the two fluorescence profiles in a double exposed picture (κ/λ distribution). An imbalanced sIgκ /sIgλ ratio of >3:1 or <1:3 was considered abnormal.

#### Cytogenetic and molecular studies. Analysis of trisomy 12, del(11q23), del(17p13.1), and del(13q14), as well as structural abnormalities involving the IgH gene were systematically investigated on FACS-purified CLL-like and CLL cells by multicolour iFISH using the following DNA probes purchased from Vysis (Downers Grove, IL): CEP12 DNA probe conjugated with spectrum orange (SO), LSI ATM (11q22.3), LSI MLL (11q23.3) dual-color probe, LSI p53 (17p13.1) conjugated with SO, LSI13/RB1 gene (13q14) conjugated with SO, LSI D13S25 (13q14.3) conjugated with SO, and LSI IgH/bcl2 t(14;18)(q32;q21) dual color probe, respectively. FISH studies were then performed on purified CLL-like / CLL B-cells fixed in 3/1 (vol/vol) methanol/acetic and hybridized. Briefly, pepsin-digested slides containing both the cells´ DNA and the probes´ DNA were denatured at 75ºC for 1 min and immediately hybridized (overnight at 37ºC), in a Hybrite thermocycler (Vysis). Then, slides were sequentially washed (5 min at 46ºC) in 50% formamide/2X sodium chloride citrate buffer (SSCb) and PBS with 1% Tween-20 (vol/vol), and counterstained with 35 µl of a mounting medium containing 75 ng/ml of DAPI (Sigma, St Louis, MO); Vectashield (Vector Laboratories, Burlingame, CA) was used as antifading agent.

High molecular weight DNA from sorted CLL-like / CLL B-cells was isolated by standard proteinase K digestion, and isopropanol precipitation in the presence of glycogen to increase the DNA yield; final washing of the DNA pellet was performed in ice cold 70% ethanol. For amplification of complete IGHVDJ rearrangements, six different family-specific VH primers and one JH consensus primer were used in one multiplex PCR reaction covering framework region (FR) 1. The primers were produced in scale (0.02 µM) and they were purified by standard HPLC (InvivoScribe Technologies, La Ciotat, France). The BIOMED-2 consortium has developed and clinically validated these primers for immune receptor amplification and their sequences were as follows: Primer name VH1/7-FR1 (5´ GGCCTCAGTGAAGGTCTCCTGCAAG-3´), Primer name VH2-FR1 (5´ GTCTGGTCCTACGCTGGTGAAACCC-3´), Primer name VH3-FR1 (5´ CTGGGGGGTCCCTGAGACTCTCCTG-3´), Primer name VH4-FR1 (5´ CTTCGGAGACCCTGTCCCTCACCTG-3´), Primer name VH5-FR1 (5´ CGGGGAGTCTCTGAAGATCTCCTGT-3´), Primer name VH6-FR1 (5´ TCGCAGACCCTCTCACTCACCTGTG-3´) and Primer name JH consensus-FR1 (5´ CTTACCTGAGGAGACGGTGACC-3´). PCR amplifications were performed using 50 or 100 ng of template genomic DNA, 10 pmol of each primer, and 0.2 µl of AmpliTaq Gold enzyme (Applied Biosystems, Foster City, CA) per 35 µl reaction. The PCR amplification used was as follows: denaturation at 95ºC for 7 min; 40 cycles at (94ºC for 30s, 59.8 ºC for 45s, 72ºC for 90s); and a final extension at 72ºC for 10 min. To obtain high-quality sequencing results, efficient post-PCR amplicon purification using ExoSAP-IT reagent (USB products, Affymetrix, Santa Clara, CA) was carried out. After ExoSAP-IT PCR clean up, the DNA was sequenced in an Applied Biosystems 3130xl Genetic Analyzer. The IgBLAST algorithm (National Center for Biotechnology Information) was used to localize those sequences which show a perfect match between forward (F) and reverse (R) reads. For each B-cell clone, we generated a consensus IGHV-D-J sequence with the matched F-R region and the correct base reads found before and after such region in the original chromatograms. Alignment of rearranged IGHV-D-J sequences to germ line V, D and J segments and determination of V-D and D-J junctions were performed using the IMGT database and tools.
